# Nationwide sustainability assessment of PFAS treatment in U.S. water utilities

**DOI:** 10.1038/s41467-026-77509-w

**Published:** 2026-10-01

**Authors:** Md. Moshiur Rahman Tushar, Zaki Alam Pushan, Christopher Sutton Page, Nirupam Aich, Lewis Stetson Rowles

**Affiliations:** 1 Department of Civil Engineering and Construction, Georgia Southern University, Statesboro, Georgia USA; 2 Department of Civil and Environmental Engineering, University of Nebraska – Lincoln, Lincoln, NE USA; 3 Department of Computer Science, Georgia Southern University, Statesboro, Georgia USA

**Keywords:** Environmental impact, Sustainability, Environmental economics

## Abstract

Per- and polyfluoroalkyl substances (PFAS) pose an unprecedented challenge for US drinking water systems as new national regulations mandate their removal. While granular activated carbon (GAC) and ion exchange (IX) resins are widely applied, their economic and environmental sustainability under diverse real-world conditions remains uncertain. Here we leverage a quantitative sustainable design framework (integrated techno-economic analysis and life cycle assessment) to evaluate GAC and IX performance across utilities nationwide. Through uncertainty and sensitivity analyses, we identify utility size, water types (e.g., groundwater and surface water), and PFAS chain length as the dominant drivers of cost and environmental impacts. IX consistently shows lower global warming potential than GAC, though treatment of short-chain PFAS significantly increases costs. Water quality, especially total organic carbon, affects treatment performance and can change cost and environmental impacts for GAC. Results highlight disproportionate burdens on small and rural systems and the trade-offs inherent in technology selection. This work provides actionable insight for utilities and policymakers, enabling more equitable and sustainable strategies for meeting emerging PFAS standards.

## Abstract (web summary)

This study assesses the techno-economic and environmental performance of granular activated carbon and ion exchange resins across U.S. water utilities, identifying key cost and impact drivers to inform more sustainable and equitable PFAS treatment strategies.

## Introduction

Per- and polyfluoroalkyl substances (PFAS) are highly persistent contaminants now regulated in US drinking water due to strong evidence of health risks^1–4^ and widespread occurrence^5–8^. In 2024, the US EPA set the first national PFAS limits^9^, with phased compliance through 2031^10^. Supplementary Section 1 provides details on regulations. These rules acknowledge that sustainable, long-term treatment strategies are essential. As public water systems mobilize to comply with these regulations, utilities face uncertainties regarding the selection of appropriate treatment technologies tailored to their specific operational contexts and site-specific PFAS profiles. Current approaches for PFAS remediation and removal broadly encompass the two categories of separation and destruction. While destruction offers the promise of permanently eliminating PFAS, significant uncertainties persist regarding their scalability, economic feasibility, and potential byproducts^11–15^. Consequently, PFAS separation currently represents a more immediate and pragmatic solution for utilities, i.e., a clearer and more actionable pathway toward regulatory compliance. Among available separation technologies, granular activated carbon (GAC) and ion exchange (IX) resins remain the most widely applied due to their established removal efficiencies for long-chain PFAS and compatibility with existing infrastructure^16–19^. However, remaining challenges for these established technologies include limited removal of short-chain PFAS, media regeneration difficulties, and operational costs^20–22^. Recently, advanced treatment methods such as reverse osmosis^23^, advanced oxidation processes^24^, and nanotechnology^25,26^ have gained attention in laboratory and pilot-scale studies. However, their broader applicability for utility-scale operations is still hindered by substantial gaps in understanding about their long-term efficacy, cost-effectiveness, and environmental sustainability^27,28^. As regulatory compliance deadlines approach, PFAS treatment choices must balance removal efficiency with cost and environmental performance, especially for systems with limited resources.

Utility size is a critical determinant of cost and sustainability, as larger plants benefit from economies of scale that reduce capital and operating costs^29^. Furthermore, tailoring design parameters and operational strategies to the specific demands of different system sizes can markedly shift the environmental profile of a treatment system^30^. Such optimization may involve changes in equipment configuration, material use, and energy demand that alter life cycle impacts. Despite their commercial deployment, GAC and IX lack comprehensive evaluations of economic and environmental sustainability under real-world conditions^31^. Recognizing the importance of utility size, the US EPA developed Work Breakdown Structure (WBS) based cost models for both GAC^32^ and IX^33^ treatment of PFAS in drinking water. However, these models do not incorporate uncertainty or sensitivity analyses, limiting their reliability when conditions deviate from assumptions. They also omit environmental trade-offs, such as changes in energy demand, material use, and waste outputs that differ across technologies and system sizes. Prior studies often suggest IX has lower costs and environmental impacts than GAC^34–38^; though outcomes vary with water quality and operating conditions. For example, GAC can generally outperform IX when treating water with high PFAS concentrations and low dissolved organic carbon^34,38^. However, existing research has not examined how these technologies perform across utilities of different sizes, leaving a critical gap in understanding their financial viability and environmental sustainability under real-world conditions. These gaps underscore the need for research that provides utilities with data-driven insights to inform cost-effective and environmentally responsible decision-making in PFAS treatment strategies.

Here, we evaluate the nationwide sustainability of GAC and IX performance for water utilities under uncertainty. Specifically, the objectives are: (i) to characterize the overall cost and environmental impacts of these technologies while incorporating uncertainty and sensitivity analyses, and (ii) to identify key sustainability drivers, with utility size categorization expected to be the most influential factor. To this end, this study leverages the quantitative sustainable design (QSD) methodology^39^, combining techno-economic analysis (TEA) and life-cycle assessment (LCA) (Supplementary Fig. 1). Trade-offs between these technologies were evaluated by considering a wide range of contextual and technological parameters. Uncertainty was systematically incorporated into the model using Monte Carlo simulations, and sensitivity analyses employing Spearman’s rank correlation coefficients were conducted to elucidate critical drivers influencing sustainability outcomes. Outcomes were evaluated across the US by quantifying cost and GWP for IX and GAC treatment of multiple PFAS compounds across utility sizes and water types (e.g., groundwater (GW) and surface water (SW)), using data from representative towns and cities in each state. This study offers evidence-based guidance to help utilities and regulators align PFAS compliance with sustainability.

## Results and discussion

### Economic and environmental sustainability of GAC and IX

**Fig. 1:**
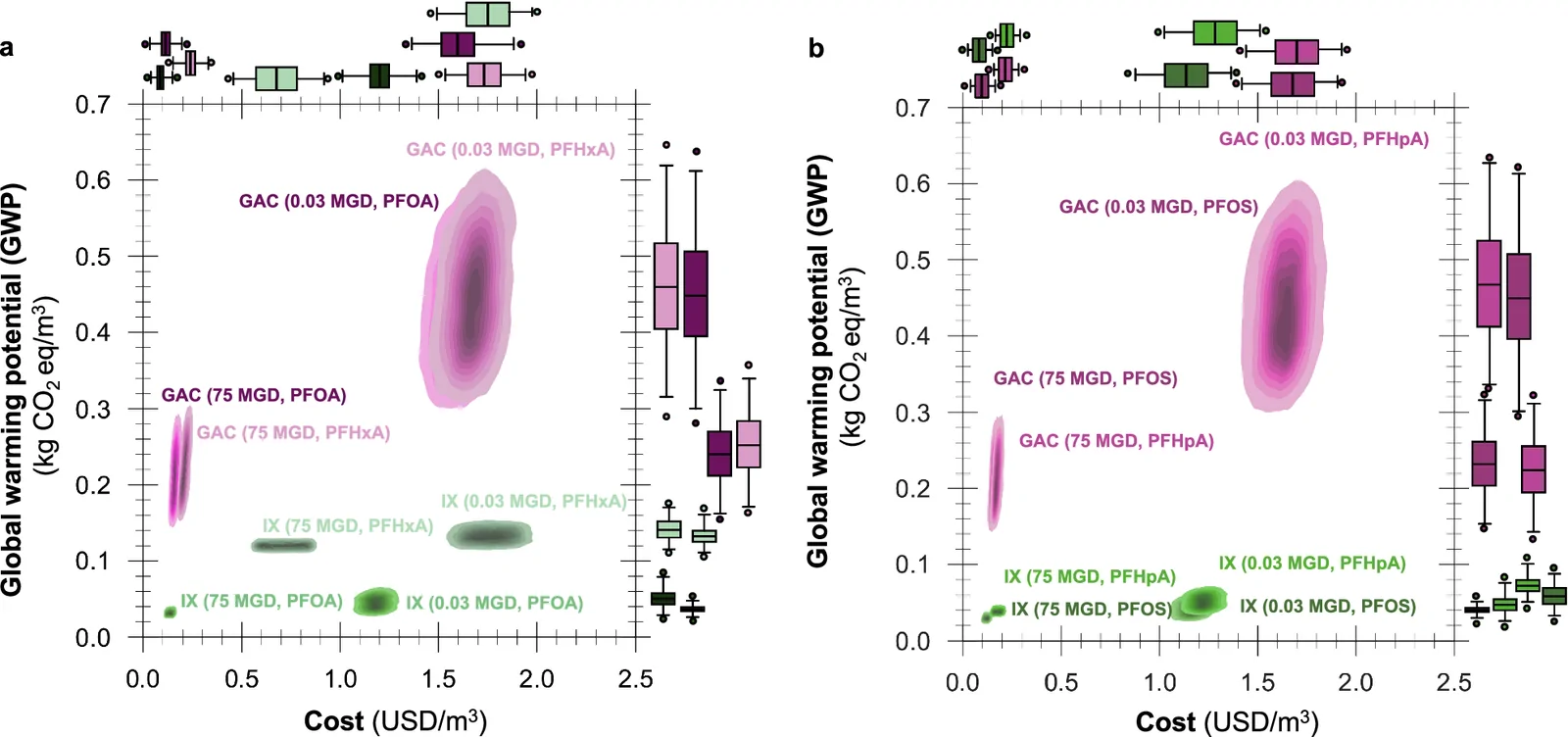
Estimates of economic and environmental outcomes associated with PFAS treatment under different technology, contaminant, and utilities. The Kernel density maps represent the distribution of estimated GWP and treatment costs for PFAS removal, based on 10,000 Monte Carlo simulations for each scenario. The horizontal axis represents treatment cost (USD/m^3^), and the vertical axis represents GWP (kg CO_2_ eq/m^3^). Box and whisker plots along the axes illustrate scenario-specific distributions of GWP and costs. This plot shows results for IX and GAC treatment of four PFAS compounds at very small (0.03 MGD) and large (75 MGD) utilities, including (**a**) PFOA and PFHxA, and (**b**) PFOS and PFHpA. Green shades represent IX treatment and violet shades represent GAC treatment, with darker and lighter shades distinguishing individual PFAS compounds within each technology. The center line of each box and whisker plot indicates the median; box edges indicate the 25th and 75th percentiles; whiskers extend to the 10th and 90th percentiles; and points represent the 5th and 95th percentiles. Source data are provided as a Source Data file.

In our first layer of analysis, we estimated the economic and environmental outcomes associated with GAC and IX technologies for PFAS treatment in drinking water utilities. We began by exploring the variability in cost and global warming potential (GWP) across different utility sizes (i.e., very small and large), water types (i.e., GW and SW), and PFAS types (i.e., perfluorooctanoic acid (PFOA) and perfluorohexanoic acid (PFHxA)). The initial analysis focused on GW conditions. We selected PFOA (long-chain, C8)^40,41^ and PFHxA (short-chain, C6)^41,42^ to compare between the PFAS with same functional groups (e.g., perfluoroalkyl carboxylic acids or PFCAs). In this study, PFHxA serves as a representative short-chain PFAS within the US EPA WBS treatment framework and provides insight into the sustainability implications of treating short-chain PFAS using GAC and IX. However, PFHxA is a relatively longer short-chain compound and should not be interpreted as representing the most challenging short-chain treatment scenario. Among perfluoroalkyl carboxylates, sorption weakens with decreasing chain length, so shorter-chain compounds such as perfluorobutanoic acid (PFBA) are expected to be more difficult to remove than PFHxA and may exhibit different sustainability trade-offs^43^. However, PFBA is not parameterized within the US EPA WBS framework and could not be evaluated here. A similar comparison was later conducted for perfluorooctane sulfonate (PFOS) (long-chain, C8)^40,41^ and perfluoroheptanoic acid (PFHpA) (shorter-chain, C7)^41,42^ to evaluate how differences in PFAS functional groups (i.e., sulfonic vs carboxylic) influence treatment sustainability outcomes (Fig. 1b).

**Fig. 2:**
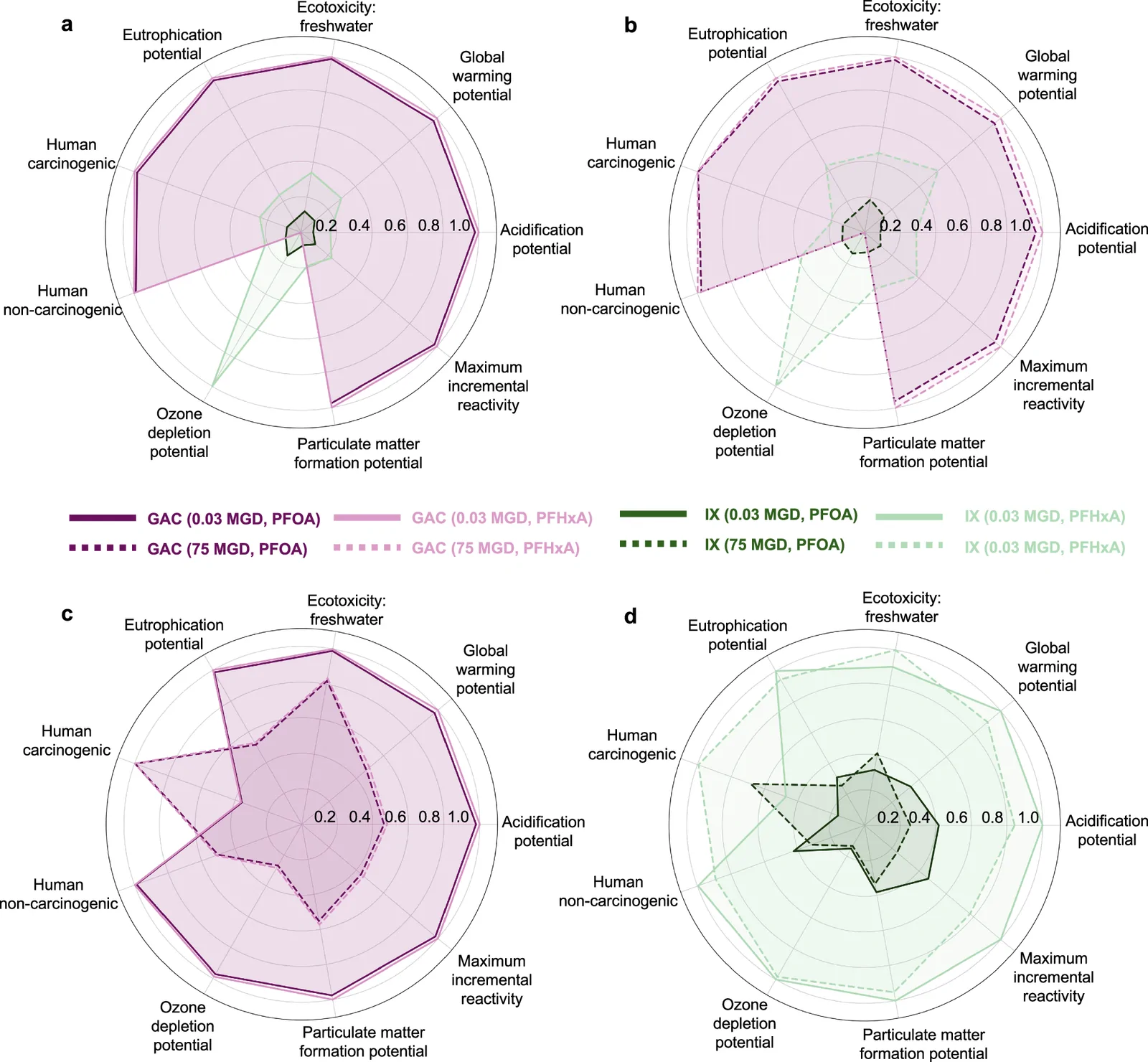
Comparative environmental trade-offs of PFAS treatment technologies and targets across system scales. Radar plots illustrate the relative performance of GAC and IX treatment for PFHxA and PFOA across nine environmental impact categories. **a** IX and GAC compared for very small-scale utilities (0.03 MGD). **b** IX and GAC compared for large-scale utilities (75 MGD). **c** PFHxA and PFOA compared for GAC treatment. **d** PFHxA and PFOA compared for IX treatment. Radar plots depict normalized impact values for each category, with larger distances from the center representing higher environmental impacts. Impact categories include acidification potential, global warming potential, ecotoxicity, eutrophication potential, human carcinogenic, human non-carcinogenic, ozone depletion potential, particulate matter formation potential, and maximum incremental reactivity. All results are normalized to per cubic meter of treated water. Source data are provided as a Source Data file.

**Fig. 3:**
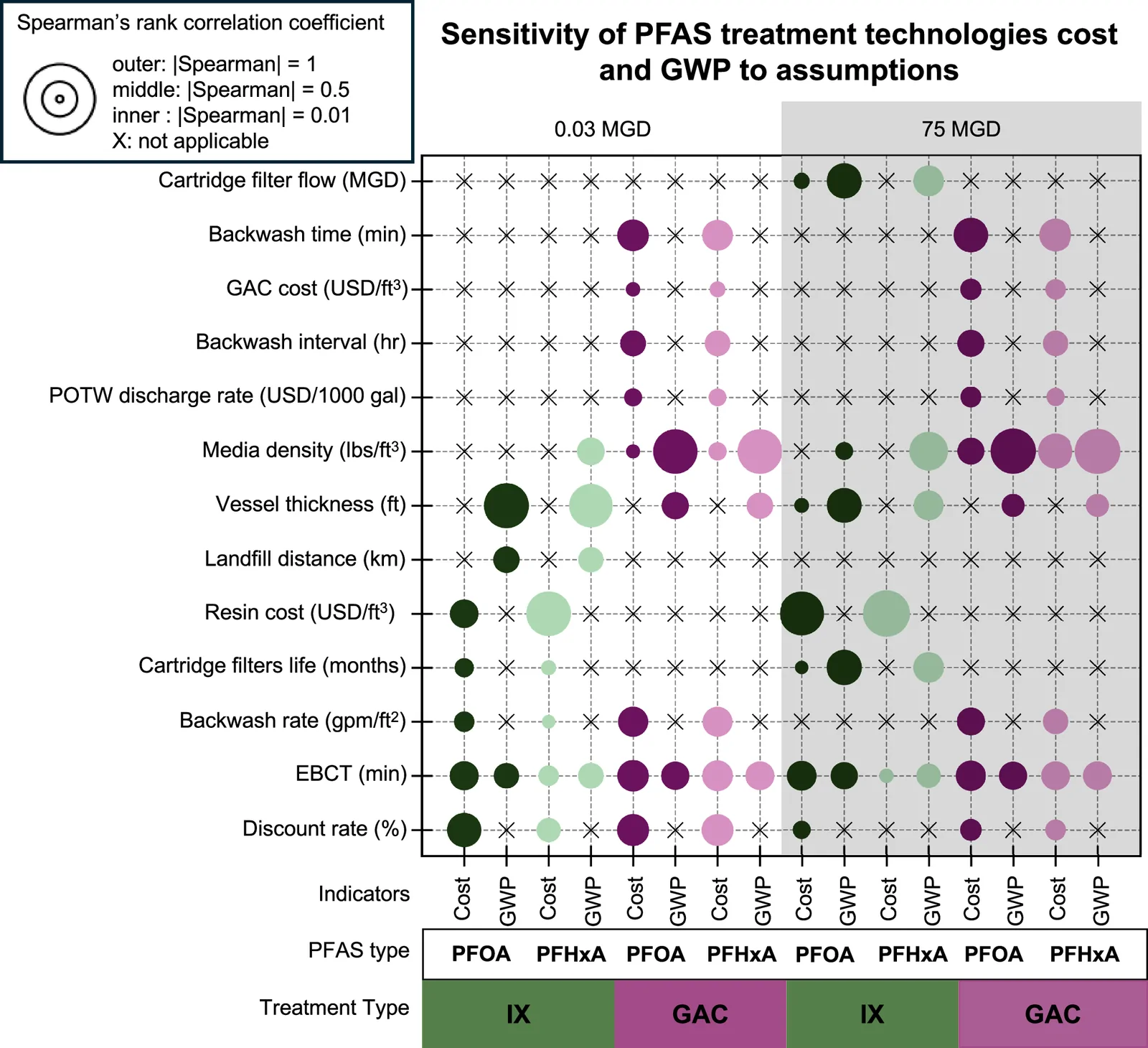
Sensitivity of PFAS treatment cost and GWP to system parameters across technologies and scales. Spearman’s rank correlation coefficients indicate the influence of key parameters on treatment cost and global warming potential (GWP) for IX and GAC treatment of PFAS-contaminated water. Parameters are shown on the ordinate, with cost and GWP for each scenario on the abscissa. Bubble size reflects the strength of the correlation, with larger bubbles indicating greater influence; marks indicate parameters not applicable to a given scenario. PFOS and PFHpA results, which follow similar trends, are presented in Supplementary Fig. 6. Source data are provided as a Source Data file.

**Fig. 4:**
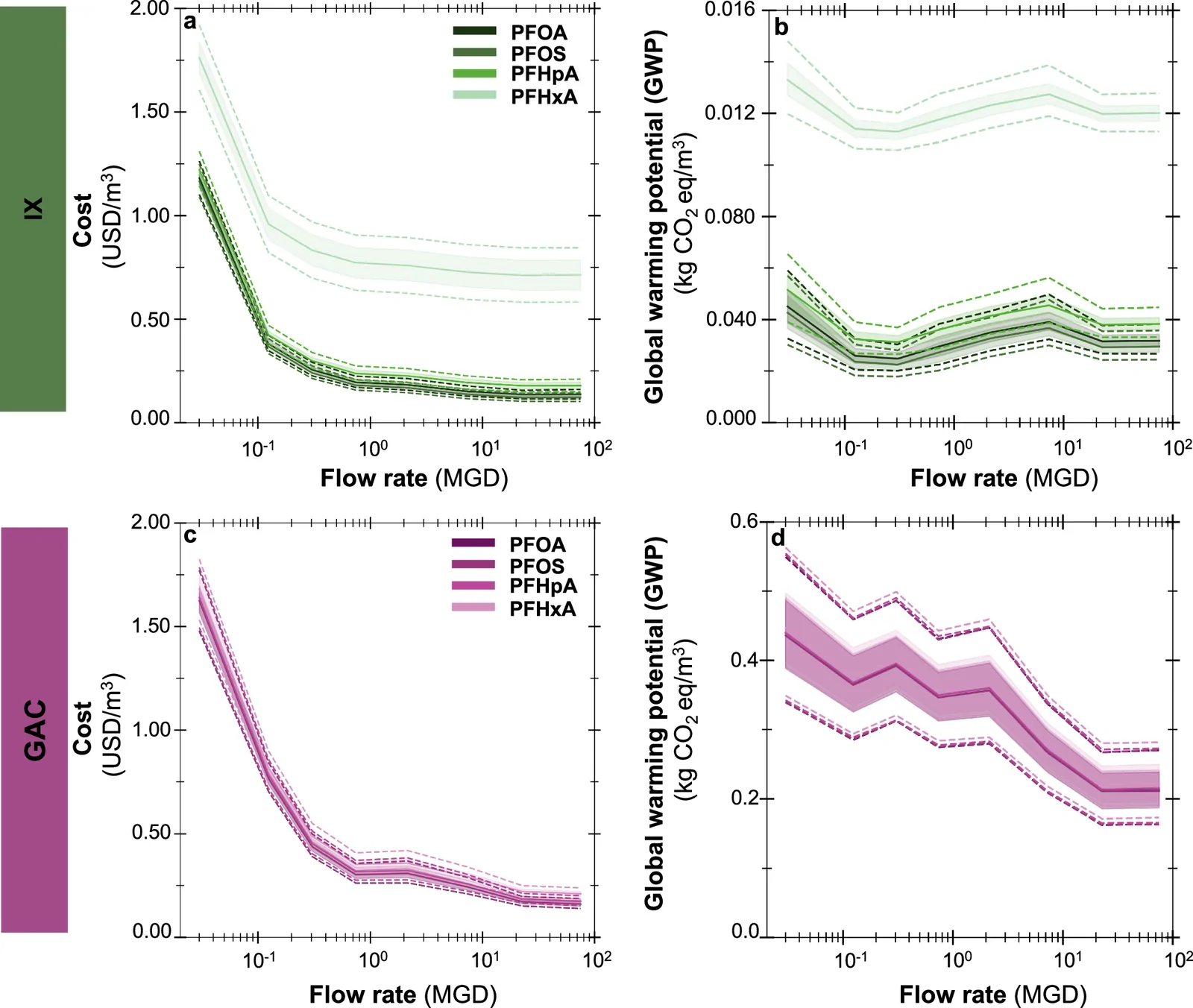
Influence of system scale on cost and GWP for PFAS treatment using IX and GAC. **a**, **b** Treatment costs and GWP for IX treatment. **c**, **d** Treatment costs and GWP for GAC treatment. Results are shown for four PFAS compounds (PFOA, PFOS, PFHpA, PFHxA) with four different shades of green (**a**, **b**) for IX and purple (**c**, **d**) for GAC across utility sizes ranging from 0.03 to 75 MGD. The median values are plotted as the center lines, shaded areas represent the 25th to 75th percentile range, and dashed lines show the 5th and 95th percentiles based on 10,000 Monte Carlo simulations. Source data are provided as a Source Data file.

While the primary analysis used a uniform 4 ng/L effluent target to provide a standardized comparison framework for GAC and IX, regulatory thresholds for several PFAS remain under active discussion and may vary across compounds and jurisdictions as time progresses. Future regulatory thresholds for short-chain PFAS (e.g., PFBS and PFHxA) may differ from those established for PFOA and PFOS. Consequently, the PFHxA results presented here (Figs. 1–4) should be interpreted as conditional on the assumed 4 ng/L treatment target. Less stringent targets would reduce media replacement requirements, and lower both costs and GWP (Supplementary Fig. 2). The costs and environmental impacts of GAC and IX for PFAS treatment vary

significantly by utility size and PFAS type (Fig. 1). Overall, very small utilities (0.03 MGD) face higher normalized costs and GWP impacts. Fixed capital and operational demands are spread over smaller flow volumes, leading to higher unit costs and less efficient use of materials and energy^44–46^. Across all scenarios, IX consistently results in lower GWP than GAC for both PFOA and PFHxA, highlighting the environmental benefits of resin-based treatment. For PFOA treatment with IX, the GWP for 0.03 MGD capacity was 0.045 kg CO_2_ eq/m^3^ (median), with a range of [0.032–0.059] kg CO_2_ eq/m^3^ [hereafter, the 5th and 95th percentile range will be shown in brackets following the median value]. For large utilities (75 MGD), the GWP declined to 0.032 [0.027–0.038] kgCO_2_ eq/m^3^. IX achieved similar reductions in GWP for PFHxA, decreasing from 0.133 [0.120–0.148] kg CO_2_ eq/m^3^ at 0.03 MGD to 0.120 [0.113–0.128] kg CO_2_ eq/m^3^ at 75 MGD. These declines reflect typical economies of scale, where fixed material and energy demands are distributed over larger water volumes.

In contrast, GAC requires a longer empty bed contact time (EBCT) than IX to achieve similar PFAS removal, which increases the needed media volume and associated embodied emissions^47^. This stems from GAC’s lower adsorption capacity and affinity for certain PFAS compared to PFAS-selective IX resins^20,47^. For PFOA treatment at 0.03 MGD, GAC produced 0.436 [0.340–0.549] kg CO_2_ eq/m^3^ (nearly an order of magnitude higher than IX), and PFHxA showed a similar pattern at 0.447 [0.349–0.563] kg CO_2_ eq/m^3^. Despite IX’s lower GWP, PFHxA treatment costs remain high: at 0.03 MGD, IX cost 1.76 [1.61–1.92] USD/m^3^ versus 1.18 [1.10–1.26] USD/m^3^ for PFOA, and at 75 MGD still costs 0.71 [0.58–0.84] versus 0.14 [0.11–0.16] USD/m^3^. IX is more expensive for PFHxA because its lower resin affinity requires greater media use or more frequent replacement^48,49^. GAC shows a similar cost gap, PFHxA requires higher treatment costs than PFOA in GAC systems, and PFHpA is slightly more costly than PFOS in both IX and GAC systems (Fig. 1b). For GW, IX offers lower emissions but higher costs for short-chain PFAS, while GAC is more cost-effective but produces higher climate impacts.

However, these trends change under SW conditions, where water quality, particularly total organic carbon (TOC), influences system performance for GAC. Results indicate that IX is largely unaffected by TOC. In contrast, GAC is highly sensitive to TOC^50–53^, leading to higher cost and GWP at elevated levels. For PFHxA, at TOC levels > 1.75 mg/L, the cost of GAC (i.e., 1.90 [1.72–2.14] USD/m^3^ at 0.03 MGD) exceeds that of IX (i.e., 1.76 [1.61–1.92] USD/m^3^ at 0.03 MGD). Under GW conditions, GAC was more cost-effective (Supplementary Fig. 3 and Supplementary Results). For PFOA removal by GAC, increasing TOC leads to higher costs and GWP compared to GW conditions. For small utilities, cost at low TOC (0.25–0.50 mg/L) is approximately 7% higher (median) than the baseline GW case (Supplementary Fig. 4a; Supplementary Results). For large utilities, cost increases by approximately four times and GWP by approximately 36% (median) at TOC levels above 2.50 mg/L compared to GW conditions (Supplementary Fig. 4b and Supplementary Results). Overall, PFAS types and water quality are critical factors in PFAS treatment for drinking water utilities, as they govern system performance and can alter the relative cost and environmental tradeoffs between GAC and IX.

### Evaluating cross-scale environmental trade-offs in PFAS treatment

In our next layer of analysis, we evaluated trade-offs across nine environmental impact categories (acidification potential, ecotoxicity, eutrophication potential, human carcinogenic, human non-carcinogenic, ozone depletion potential, particulate matter formation potential, maximum incremental reactivity, and GWP) to assess the broader sustainability of PFAS treatment systems (Fig. 2). IX showed lower normalized impacts than GAC in eight of nine categories for both very small (Fig. 2a) and large (Fig. 2b) utilities, highlighting the environmental advantage of resin-based treatment. The only exception was ozone depletion potential, where IX showed higher impacts, especially for PFHxA, likely from emissions of ozone-depleting compounds during polymer synthesis and amination in resin production^34,36^. Beyond treatment technology, system size and the specific PFAS targeted further influence environmental outcomes across categories.

System size strongly influenced environmental performance. Specifically, smaller utilities showed higher normalized impacts across seven categories, particularly for IX (Fig. 2d), reflecting higher material and energy consumption per cubic meter of treated water. An exception was human carcinogenic and ecotoxicity, where very small IX systems (0.03 MGD) had lower impacts, possibly due to different waste management efficiencies or lower energy recovery burdens. GAC showed similar size-related patterns (Fig. 2c), though large systems had higher human carcinogenic impacts, likely from emissions or exposure pathways during large-scale carbon production and transport.

PFAS type also shaped outcomes. PFHxA consistently produced higher impacts than PFOA in IX across all nine categories, reflecting its lower adsorption affinity, earlier breakthrough, and greater resin demand^47,54^. In contrast, GAC showed minimal differences between PFHxA and PFOA, as the higher EBCT required for both reduces sensitivity to PFAS chain length^43,47^. These results underscore the importance of matching treatment choice to the PFAS profile of source waters, as short-chain PFAS can reduce the environmental efficiency of IX systems that perform well for long-chain compounds. Together, these findings show that sustainable PFAS treatment depends on considering system scale, PFAS properties, and technology-specific trade-offs across multiple environmental dimensions.

### Elucidating key drivers of cost and GWP

We next identified the key drivers of PFAS treatment cost and GWP by breaking down system components and conducting a global sensitivity analysis (141 IX and 146 GAC parameters). Capital, energy, and operation and maintenance (OM) contributions were quantified to show the percentage of total cost and GWP per cubic meter for each scenario (Supplementary Fig. 5). Across all scenarios, direct media costs were the dominant economic drivers (Fig. 3), with resin and GAC media representing the primary material costs^55^. This reflects the material-intensive nature of both technologies, where media procurement substantially contributes to life-cycle costs. Media density also strongly influenced outcomes, especially at larger scales, where higher total material demand amplified its effect on cost and environmental performance.

EBCT showed positive correlations with both cost and GWP, as longer EBCT increases vessel size and media requirements, driving up material use, energy consumption, and overall system impacts^20^. However, shortening EBCT does not necessarily reduce media demand or energy use, as lower EBCT can reduce adsorbent utilization and increase media requirements depending on PFAS properties and water quality^20^. Selecting an optimal EBCT requires aligning PFAS characteristics (e.g., chain length, functional group), media density, and source water quality to balance economic and environmental performance.

System sensitivity also shifted with size. Large utilities (75 MGD) showed fewer dominant parameters, indicating greater stability, while very small systems were influenced by more parameters. For GAC, backwash time negatively correlated with cost, especially at large scales, suggesting that optimizing backwash can reduce material losses and operational expenses. Contextual factors such as vessel thickness were more influential at very small scales, where infrastructure accounts for a larger share of impacts. For IX, cartridge filter life and flow negatively correlated with both cost and GWP, as extending filter life reduces replacement frequency, material use, and operational costs, with the strongest effects at very small scales. At large scales, filter performance had a greater influence on GWP, especially for PFOA, reflecting life-cycle emissions from filter production and disposal.

Landfilling is the primary end-of-life option for spent GAC and IX resins from PFAS treatment, requiring transport to permitted PFAS-capable landfilling facilities. At very small scales, landfill distance significantly increases GWP for IX because low waste volumes magnify transport emissions, while this effect is minimal at larger scales. Overall, PFAS treatment sensitivity depends on decision variables and key technological and contextual factors, and improving material efficiency, filter longevity, and backwash operations can lower both costs and environmental impacts.

### Impact of system size and PFAS type on sustainability

This analysis expanded from two PFAS (PFOA, PFHxA) at very small and large utilities to all four target PFAS (PFOA, PFOS, PFHpA, PFHxA) across eight flow rates, spanning 0.03-75 MGD (Fig. 4). This work enabled evaluation of how cost and GWP scale across PFAS types covering both carboxylic and sulfonic groups and short- versus long-chain compounds. Smaller utilities experienced disproportionately higher costs and impacts, driven by economies of scale under consistent design and operational assumptions. At very small scales, costs were highest across all PFAS and technologies, while large systems had much lower values, illustrating the scale-sensitive nature of treatment systems. PFAS type strongly affected IX performance. PFHxA consistently had higher costs and GWP, exceeding PFOA values by over fivefold for cost and nearly fourfold for GWP at large scales, reflecting its lower sorption affinity and frequent media replacement needs^43,54^. In contrast, GAC showed relatively stable cost and GWP across PFAS types, with only modest PFHxA increases.

Costs generally declined with increasing flow for both technologies but showed nonlinear shifts. For GAC, costs rose slightly ( ~ 0.01 USD/m^3^) between 0.74 and 2.152 MGD as GAC demand jumped from 41,755 lb to 150,296 lb, a 260% increase despite only a 190% flow increase (Fig. 4c). This shift reflects WBS model design transitions (e.g., addition of fiberglass storage tanks^32^). At 2.152 MGD, costs reached 0.31 [0.26-0.37] USD/m^3^ for PFOA and PFOS, 0.32 [0.28-0.38] for PFHpA, and 0.36 [0.31-0.42] for PFHxA. By 75 MGD, GAC costs declined to 0.16 [0.13–0.18] for PFOA, 0.16 [0.14-0.19] for PFOS, 0.17 [0.15-0.20] for PFHpA, and 0.21 [0.18–0.24] USD/m^3^ for PFHxA. IX did not show a cost spike at 2.152 MGD, but cost reductions slowed, likely from similar design shifts. At 75 MGD, IX costs were 0.14 [0.11-0.16] USD/m^3^ for PFOA, 0.12 [0.10-0.14] for PFOS, 0.18 [0.15-0.21] for PFHpA, and 0.71 [0.58-0.84] USD/m^3^ for PFHxA.

For IX, GWP declined overall with scale but had spikes linked to design transitions (Fig. 4b). Between 0.305 and 0.740 MGD, PFHxA GWP rose from 0.113 [0.106-0.120] to 0.118 [0.109-0.128] kg CO_2_ eq/m^3^, likely from switching fiberglass vessels to denser carbon steel^33^. From 0.740 to 2.152 MGD, GWP rose sharply as resin scaled inefficiently, backwash pumps were added, and cartridge filters doubled from 4 to 8 (high-impact polymers/stainless steel). Another increase occurred at 7.365 MGD (0.123 → 0.127 kg CO_2_ eq/m^3^) when PVC check valves were replaced by heavier cast iron. Beyond this size, values stabilized from 22.614-75 MGD.

GAC showed a more irregular GWP pattern (Fig. 4d). After an initial decline, GWP rose between 0.124 and 0.305 MGD with the fiberglass-to-steel vessel transition, then decreased at 0.740 MGD before rising again at 2.152 MGD, mirroring IX patterns. Beyond this point, GWP declined steadily as scale increased, reflecting improved efficiency in medium and large systems. Together, these results reveal strong non-linear relationships between system size, cost, and GWP, highlighting how design transitions and scale efficiencies shape PFAS treatment sustainability.

### Evaluating location-specific outcomes of PFAS treatment technologies nationwide

**Fig. 5:**
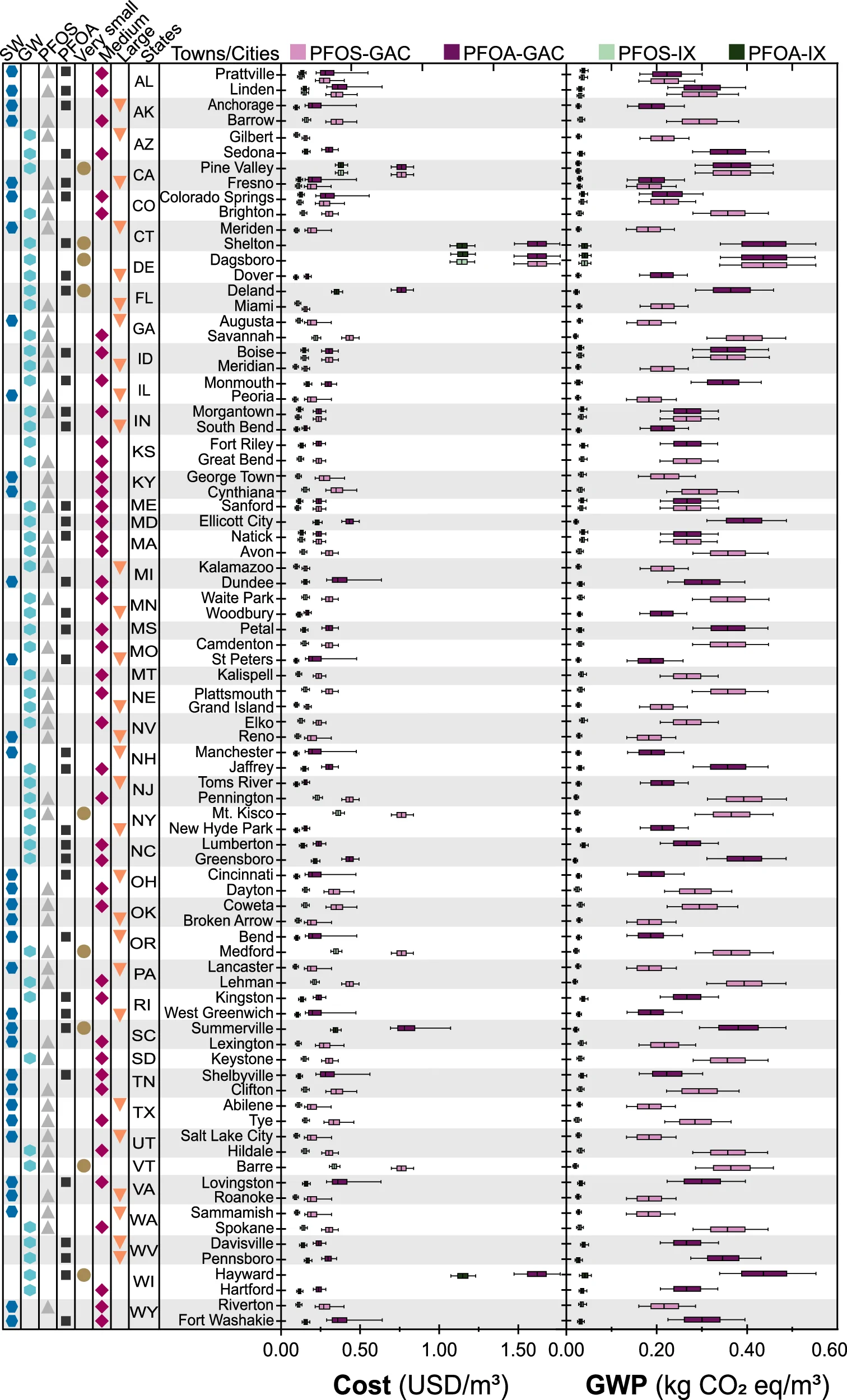
City-level variation in PFAS treatment cost and GWP across the United States based on measured contamination levels. The figure shows treatment cost and GWP for IX and GAC systems deployed to address observed exceedances of regulated PFOA and PFOS concentration (4 ng/L) across US cities. Results are organized by PFAS type, utility size (i.e., very small, medium, and large), water type (i.e., GW and SW), state, and town/city. Six states (New Mexico, North Dakota, Louisiana, Iowa, Hawaii, and Arkansas) are excluded as no cities reported PFAS concentrations exceeding regulatory thresholds. All other states contain one or more cities with PFOA or PFOS levels above the regulated limit. Box and whisker plots represent the distribution of cost and GWP for each city, illustrating the economic and environmental burdens municipalities may face under current contamination patterns. The center line of each box and whisker plot indicates the median; box edges indicate the 25th and 75th percentiles; whiskers extend to the 10th and 90th percentiles; and points represent the 5th and 95th percentiles. Supplementary Table 1 provides PFAS occurrence, utility size, and water source (GW or SW) across U.S. drinking water systems. Source data are provided as a Source Data file.

We assessed GAC and IX treatment costs and GWP for meeting current PFOA and PFOS target concentration (4 ng/L) across 82 drinking water systems in the US, while explicitly incorporating water source effects (Fig. 5). Among these systems, 34 are supplied by SW and 48 by GW. For SW systems, TOC was varied within a representative range of 0.5–2.7 mg/L to capture its influence on GAC performance, as site-specific TOC data were not available, and this range reflects typical SW conditions within the validity of the model^56,57^. Overall, IX generally showed lower cost and GWP than GAC across most systems. This difference was largely attributable to higher TOC levels in utilities using SW, which increased variability in both cost and GWP estimates.

Very small utilities showed the highest normalized burdens. For GW systems,

Shelton, CT (72 customers, 5.6 ng/L PFOA) had the highest estimated treatment burdens when using GAC with costs of 1.63 [1.48–1.77] USD/m^3^ and GWP of 0.436 [0.341–0.552] kg CO_2_ eq/m^3^. For SW systems, the presence of TOC further increased GAC cost compared to IX. For example, Summerville, SC (275 customers, 5.2 ng/L PFOA) represents the highest GAC cost under SW conditions, reflecting the strong influence of organic matter on the treatment capacity of GAC. In contrast, IX remains less affected by the water source. For example, Hayward, WI (57 customers, 7.3 ng/L PFOA) had the highest IX values at 1.15 [1.08–1.24] USD/m^3^ and 0.041 [0.029–0.055] kg CO_2_ eq/m^3^. These results align with earlier findings that limited economies of scale and frequent media replacement drive high costs and GWP in very small systems, with additional increases for GAC in SW systems.

Larger systems had far lower normalized burdens; however, similar trends on the influence of water source on GAC performance were observed. For Reno, NV (442,000 customers, 13 ng/L PFOS, SW), the estimated GAC cost was 0.19 [0.15–0.32] USD/m^3^, whereas Miami, FL (2.3 million served, 14 ng/L PFOS), supplied by GW, showed a lower cost of 0.16 [0.14–0.18] USD/m^3^ despite its largest size in the dataset and similar PFAS conditions. Peoria, IL (137,575 customers, 4.1 ng/L PFOS) had the lowest IX cost at 0.09 [0.08–0.11] USD/m^3^. Meriden, CT (58,441 customers, 7.9 ng/L PFOS, SW) had the lowest GAC GWP at 0.210 [0.162–0.268] kg CO_2_ eq/m^3^, while Lehman, PA (875 customers, 5.2 ng/L PFOS, GW) had the lowest IX GWP at 0.019 [0.014–0.025] kg CO_2_ eq/m^3^. These GWP variations reflect design parameters in the WBS model rather than a simple link to system size. PFAS concentration plays a secondary role compared to utility size and water quality. Pine Valley, CA (181.4 ng/L PFOA, GW) had moderate IX values of 0.38 [0.35–0.43] USD/m^3^ and 0.026 [0.021–0.033] kg CO_2_ eq/m^3^ and GAC values of 0.77 [0.70–0.84] USD/m³ and 0.365 [0.285–0.458] kg CO_2_ eq/m^3^, well below costs seen in small systems like Shelton or Hayward. In contrast, Peoria’s very low concentration (4.1 ng/L PFOS) still produced the lowest IX cost, driven by its large utility scale (137,575 customers). Cincinnati, OH (750,200 served, PFOS ~ 1.3x limit, SW) showed higher GAC costs compared to IX, with IX at 0.10 [0.09–0.12] USD/m^3^ and GAC at 0.20 [0.16–0.48] USD/m^3^. This contrast indicates that water quality can reduce or eliminate the cost advantage of GAC observed under GW conditions. Although these large systems had lower impacts than small communities, they were not the absolute lowest due to differences in water types, PFAS types and levels. Sensitivity analysis of influent PFAS concentration (20–1000 ng/L) showed IX costs varied only 1–12% and GWP 5–8% (Supplementary Fig. 7), while utility size exerted far greater influence. However, the inclusion of water quality, particularly TOC for GAC, introduces additional variability and can shift the relative performance of treatment technologies across locations. Additional analysis was conducted to evaluate how different effluent PFAS targets affect treatment sustainability (Supplementary Fig. 2). Increasing the allowable effluent concentration from 4 to 24 ng/L reduced treatment costs by 6.25–21.35% for GAC and 24.37–46.23% for IX, while GWP decreased by 2.99–8.97% for GAC and 22.48–54.39% for IX at 75 MGD for PFHxA. For PFHxA, less stringent effluent targets substantially reduced media replacement demand and associated environmental burdens, whereas PFOA showed comparatively smaller changes due to its stronger sorption affinity and longer bed life. These results suggest that future regulatory thresholds may strongly influence treatment sustainability, especially for utilities treating lower-affinity short-chain PFAS.

While this study uses US-based regulatory conditions as a reference, PFAS contamination and management are global challenges with region-specific priorities. To improve the general applicability of the QSD model and address variability in treatment performance across different media, PFAS mixtures, and water quality conditions, a generalized framework is proposed (Supplementary Fig. 8). This framework links pilot-scale performance data to system-level cost and environmental impact estimation. Supplementary Section 3 provides a detailed description of the framework and its implementation. The proposed framework is not limited to the conditions evaluated here and can be applied in any region by incorporating local pilot-scale performance data. Once site-specific treatment performance is available, the framework can be used to estimate cost and environmental impacts under local regulatory targets, water quality, PFAS mixtures, and energy systems. For instance, application of the framework using literature-derived variability in Freundlich parameters (K and 1/n) for multiple GAC systems demonstrated measurable differences in system-level cost and GWP, even for the same GAC media under different treatment configurations and water quality conditions (Supplementary Section 3 and Supplementary Table 2). Geographic differences also suggest that future PFAS contamination could create highly uneven impacts across US communities, despite some states (NM, ND, LA, IA, HI, AR) currently reporting no exceedances.

### Limitations

In this study, media bed life was estimated using US EPA equations^47^, which account for TOC (GAC) and inorganic anions (IX) but are based on limited pilot/full-scale studies primarily using GW^21,58–60^. The GAC model is validated only up to 2.8 mg/L TOC, and the IX model up to 7044 ng/L influent PFAS^47^. This reliance on GW data is a key limitation, as SW are typically more variable in composition and may produce higher costs and impacts than estimated here.

EPA WBS treatment models to help the utilities focused in achieving compliance with the current regulatory limits and therefore does not capture the full diversity of PFAS chemistries. While the selected compounds represent both carboxylate and sulfonate functional groups as well as long- and short-chain PFAS, additional compounds may exhibit different treatment behaviors. In particular, PFAS with shorter chain lengths (e.g., PFBA^43^) than those evaluated in this study and ultrashort-chain PFAS (e.g., trifluoroacetic acid (TFA)^42^) are generally more difficult to remove using sorption-based technologies and may exhibit different sustainability trade-offs than those reported here. Future research should expand sustainability assessments to additional PFAS compounds to improve understanding of treatment performance, costs, and environmental impacts across a broader range of PFAS chemistries and regulatory scenarios. Nevertheless, the findings provide valuable insights for utilities currently making PFAS treatment decisions under the evolving US regulatory landscape.

This study focuses on single-use GAC and IX, reflecting current practice where regeneration or reactivation is often not implemented due to performance limitations and operational complexity in drinking water treatment^58^. Future work should evaluate the feasibility and sustainability of GAC reactivation and IX regeneration at utility scale, which could affect costs and embodied emissions. End-of-life management also remains underexplored. This study assumed landfill as the primary disposal pathway for spent media. Alternative disposal options, such as incineration, may lead to different costs and environmental outcomes and should be evaluated in future studies.

### Implications

Overall, this study elucidates key dimensions of sustainability in PFAS treatment by integrating techno-economic and life-cycle perspectives through a quantitative sustainable design framework. IX generally has lower costs and environmental impacts than GAC, though short-chain PFAS remain costly to treat. In addition, GAC can provide additional treatment benefits by removing TOC and a wide range of trace organic contaminants, while PFAS-selective IX resins are designed for targeted PFAS removal^61^. As organic matter competes with PFAS for adsorption, PFAS treatment should be placed toward the end of the treatment train in high-TOC waters to improve performance. Utility size was identified as the overarching driver of economic and environmental performance, though contextual parameters (e.g., PFAS type) and technological parameters (e.g., media density) also influenced outcomes. Small and rural systems face disproportionate burdens, highlighting the need for targeted policy and financial support to prevent widening inequities in safe water access. These findings underscore the need to select PFAS treatment technologies that are appropriate for utility size and highlight opportunities to address critical barriers facing smaller systems. Framing PFAS remediation as a sustainability challenge shifts the conversation from compliance to resilience. These insights provide a foundation for designing treatment strategies that safeguard public health and support more informed decision-making.

## Methods

### PFAS treatment technologies

To explore trade-offs among GAC and IX, we leverage the QSD methodology where integrated TEA and LCA were performed with parameters covering design, materials, energy, capital requirements, and OM requirements (Supplementary Fig. 1)^39^. The selection of appropriate media plays a critical role in the sustainability performance of PFAS treatment systems, as media properties directly influence removal efficiency and replacement frequency. In this study, we focused on single-use IX resin and GAC systems, aligning with prevailing practice for drinking water applications where regeneration is generally not recommended due to performance limitations and operational complexity^32,33^. For both treatment technologies, we adopted a throwaway management strategy, consistent with current utility-scale deployment scenarios. Spent backwash was discharged to a publicly owned treatment works, while spent resin and GAC were assumed to be disposed via landfill as the final end-of-life pathway. To reflect state-of-the-art practice, we selected PFAS-selective IX resins, which have been specifically engineered to enhance removal efficiency across a broad spectrum of PFAS compounds. These resins offer superior adsorption capacity and are particularly well-suited for single-use configurations where spent media is replaced, rather than regenerated-an approach supported by pilot studies and vendor recommendations^62–64^. For IX, the foundational rapid small-scale column test (RSSCT) dataset consists of two commercially available PFAS-selective resins (Purofine PFA694E and Dowex PSR-2) evaluated under a range of GW conditions, and for GAC, the US EPA considered multiple peer-reviewed studies under varied water quality conditions and represents a performance envelope reflecting best-performing carbons, with TOC explicitly incorporated as a governing parameter^58,60,65–67^. Additional details on the IX resins and GAC datasets used by the US EPA are provided in Supplementary Section 2 and Supplementary Table 3–5. For this study, all key decision variables, along with contextual and technological parameters, were primarily drawn from the US EPA’s cost estimation framework, supplemented by data from peer-reviewed studies, manufacturer specifications, and technical guidance documents. Full parameter lists and data sources for both IX and GAC systems are provided in Supplementary Information (Supplementary Data 1 and Supplementary Data 2). To improve the general applicability of the QSD model and address variability in treatment performance across different media, PFAS mixtures, and water quality conditions, a generalized framework is proposed (Supplementary Fig. 8). This framework links pilot-scale performance data to system-level cost and environmental impact estimation. Supplementary Section 3 provides a detailed description of the framework and its implementation. This framework integrates site-specific system characterization with pilot-scale testing or RSSCT-derived media performance data to generate key design inputs, particularly bed life, under representative water quality, PFAS composition, treatment target, and technology conditions. These inputs are then used in the QSD framework to evaluate both treatment costs and environmental impacts, enabling flexible assessment of different GAC products, IX resins, PFAS mixtures, and water quality scenarios without relying solely on generalized bed-life relationships. The sustainability assessment, along with uncertainty and sensitivity analyses, was carried out in Python using custom-built scripts. To support transparency and reproducibility, all modeling scripts and underlying assumptions used in this study are openly accessible via GitHub^68,69^.

#### Targeted PFAS contaminants

PFAS compounds evaluated in this study were selected based on the availability of compound-specific bed-life relationships within the US EPA WBS treatment models, which provide the engineering basis for technology sizing, cost estimation, and sustainability assessment^47^. This study initially studied six PFAS compounds: PFOA, PFOS, PFHpA, PFHxA, perfluorobutane sulfonic acid (PFBS), and perfluorohexanesulfonic acid (PFHxS). However, PFBS and PFHxS were not included in the final analysis because they do not provide additional treatment-performance information under the WBS models. For GAC, PFBS, PFHpA, and PFHxS share identical bed-life relationships, while for IX, PFBS, PFOS, and PFHxS share identical bed-life relationships^47^. To ensure that the selected PFAS span meaningful variability in treatment behavior, compound selection was guided by the US EPA bed life equations^47^ (Supplementary Section 2). Consequently, PFOA, PFOS, PFHpA, and PFHxA were selected to represent the full range of distinct treatment behaviors available within the EPA framework while avoiding redundant simulations. Together, these four PFAS were selected to examine how differences in functional group chemistry and chain length influence treatment performance and sustainability outcomes. PFOA and PFHpA are carboxylic acids, while PFOS and PFHxA represent sulfonic and shorter-chain carboxylic acids, respectively. These compounds were also selected in alignment with the US EPA’s first-ever national drinking water regulation for PFAS, which establishes legally enforceable maximum contaminant levels (MCL) and reflects mounting concern over their persistence, toxicity, and widespread occurrence in US water systems. PFOA and PFOS, both long-chain compounds, are among the most studied and historically widespread, and their inclusion in the US EPA’s MCLs (4 ng/L each) reflects their high toxicity and persistence. PFHxA and PFHpA, both short-chain carboxylates, are increasingly used as replacements for phased-out long-chain PFAS, raising concern due to their mobility and resistance to conventional treatment^70^. To maintain consistency across simulations and evaluate treatment performance under regulated conditions, we assumed an initial concentration of 100 ng/L for each compound and a final concentration of 4 ng/L, in accordance with the US EPA’s enforceable MCL thresholds for PFOA and PFOS, whereas PFNA, PFHxS, HFPO-DA (GenX), and PFBS are regulated under a hazard index framework^58^. The uniform 4 ng/L endpoint used here serves as a standardized modeling target to enable direct comparison of treatment sustainability across compounds, rather than implying identical regulatory limits for all PFAS. This selection enables a policy-relevant assessment of treatment sustainability across a chemically diverse subset of PFAS, offering utilities practical insight into how different compound classes may respond to removal technologies under the constraints of the new regulatory standards.

### System size classification

To capture the broad diversity of public water systems, we adopted eight system size categories defined by the US EPA’s drinking water treatment technology unit cost estimation model, which classifies systems based on population served and corresponding design flow rates. Design flows for these categories range from 0.030 million gallons per day (MGD), representing very small systems serving fewer than 100 people, to 75.072 MGD, capturing the needs of large urban utilities serving over 100,000 people^32,33^. This range allows for a systematic assessment of treatment performance and sustainability across utility scales, enabling insights into how system size influences cost and environmental trade-offs. By aligning system sizes with US EPA-established classifications, our study supports utility decision-making in both small and large communities, helping them navigate regulatory compliance and investment planning under the new PFAS standards.

### Techno-economic analysis (TEA)

To evaluate the economic sustainability of PFAS treatment systems, we leveraged TEA of GAC and IX technologies. Costs were estimated separately for capital, OM, and energy components, which were then aggregated to determine the total cost per unit flow. All results were normalized to USD/m^3^ to enable consistent comparison across technologies and system sizes. Capital costs reflect the initial investment required to install treatment infrastructure, while OM captures recurring expenses such as media replacement, labor, and other consumables. Energy use was treated as a distinct category to clarify its contribution to total financial sustainability. All costs were annualized over a 20-year system lifetime using discounted cash flow analysis with a 5% discount rate^71^, capturing the time value of money and smoothing expenditure across the life cycle. While this approach allows for comparability, actual expenditures are not evenly distributed over time-capital costs are typically concentrated in the early years, while OM and component replacement costs emerge throughout the system’s operation. To ensure a consistent system boundary, the analysis excludes costs assumed to be equivalent across both technologies, including land acquisition, buildings, HVAC, and energy demand for lighting, cooling, or water transfer. (Supplementary Fig. 9). By isolating the key cost drivers specific to GAC and IX, this analysis provides utilities with a more actionable understanding of long-term investment and operational trade-offs under the new PFAS regulatory landscape.

### Environmental life cycle assessment (LCA)

To assess the environmental sustainability of GAC and IX treatment systems, we conducted an LCA that captured impacts associated with capital inputs, OM requirements, and energy consumption. The analysis employed the US EPA’s TRACI v2.1 (Tool for the Reduction and Assessment of Chemicals and Other Environmental Impacts) methodology, which characterizes emissions across nine impact categories: global warming potential (kg CO₂-eq), acidification potential (kg SO_2_-eq), eutrophication potential (kg N-eq), freshwater ecotoxicity (CTUe), human toxicity-carcinogenic and non-carcinogenic (CTUh), ozone depletion potential (kg CFC-11-eq), particulate matter formation (kg PM_2.5_-eq), and maximum incremental reactivity (kg O_3_-eq). Emissions and resource use data for each system component were sourced from the ecoinvent v3.9.1 database. Impact assessment was subsequently carried out to translate inventory data into environmental outcomes, with particular attention given to GWP as a key indicator of climate-related sustainability. To better distinguish energy-related impacts from material-related burdens, energy use was treated separately from OM, allowing for a clearer understanding of its specific contribution to overall environmental performance. This disaggregated approach enables a more targeted interpretation of sustainability trade-offs and informs opportunities for impact reduction in future system design and deployment.

### Uncertainty and sensitivity analysis

To account for uncertainty in model inputs and assumptions, variability ranges were assigned to each parameter in the analysis based on data availability and confidence levels^72–75^. When only a single point estimate was available, a uniform distribution with a bounded range (typically ± 25%, unless otherwise justified) was applied to represent uncertainty. When limited published bounds or 2–20 observations were available, uniform distributions were defined using reported lower and upper values, with optional expansion of the range by 10–25% where appropriate to account for additional variability. For parameters with sufficient data ( ≥ 3 observations or defined minimum, mean, and maximum values), triangular distributions were used (e.g., EBCT) to better represent central tendency and variability. A total of 10,000 Monte Carlo simulations were performed to incorporate uncertainty^76^. This process produced a distribution of outcomes for each scenario, from which the median, 5th percentile, and 95th percentile values were extracted to characterize the central tendency and uncertainty range reported in the results. To identify key drivers influencing system sustainability, we conducted a sensitivity analysis using Spearman’s rank correlation coefficients. This non-parametric approach quantified the relationship between input parameters and model outputs across all simulations. Only parameters with statistically significant correlations (*p*-value < 0.05) and absolute coefficient values exceeding |0.05| were retained, highlighting the key drivers of cost and environmental impacts in PFAS treatment systems.

## Supplementary information

Supplementary Information

Description of Additional Supplementary File

Supplementary Data 1

Supplementary Data 2

Transparent Peer Review file

## Source data

source data

## Data Availability

All other relevant data are available from the corresponding author on request. The data supporting the findings of this study are available within the article and its Supplementary Information. Source data for all figures are provided with this paper.

## Code availability

All custom scripts used for the techno-economic analysis, life cycle assessment, and uncertainty and sensitivity analyses in this study are available on GitHub at https://github.com/lsrowles/PFAS_water_utilities^68,69^.
