# Supplementary material for: Nationwide sustainability assessment of PFAS treatment in U.S. water utilities
Source: Nat Commun. 2026 Oct 1;17:11276. doi: 10.1038/s41467-026-77509-w (PMC13720099; doi:10.1038/s41467-026-77509-w)
Supplement: Supplementary file 1 [file 41467_2026_77509_MOESM1_ESM.pdf]

Supplementary Information for

Nationwide Sustainability Assessment of PFAS Treatment in U.S. Water Utilities

Md. Moshiur Rahman Tushar[1]; Zaki Alam Pushan[2]; Christopher Sutton Page[3]; Nirupam Aich[2*]; Lewis Stetson Rowles[1*]

[1]Department of Civil Engineering and Construction, Georgia Southern University, Statesboro, Georgia 30458, United States

[2]Department of Civil and Environmental Engineering, University of Nebraska – Lincoln, Lincoln, NE, USA

[3]Department of Computer Science, Georgia Southern University, Statesboro, Georgia 30458, United States

Corresponding authors: Lewis S. Rowles, email: lrowles@georgiasouthern.edu; Nirupam Aich, email: nirupam.aich@unl.edu

Table of Contents (32 total pages):

Supplementary Figure 1. Quantitative sustainable design (QSD) methodology 5
Supplementary Figure 2. Influence of effluent PFAS concentration on cost and GWP for PFAS treatment using IX and GAC at 75 MGD 6
Supplementary Figure 3. The Kernel density maps for IX and GAC treatment of PFHxA under different TOC levels 7
Supplementary Figure 4. The Kernel density maps for IX and GAC treatment of PFOA under different TOC levels 8
Supplementary Figure 5. Baseline scenarios broken down by unit processes for PFOS treatment using GAC and IX 9
Supplementary Figure 6. Spearman's rank correlation for cost and GWP for GAC and IX systems treating PFOA, PFOS, PFHpA, and PFHxA 10
Supplementary Figure 7. Influence of flow rate and PFOA concentration on treatment cost and GWP for IX and GAC technologies 11
Supplementary Figure 8. Framework for linking pilot-scale performance to cost and environmental impacts of PFAS treatment using QSD model 12
Supplementary Figure 9. System boundaries and foreground inventory for two drinking water treatment systems for PFAS removal 13
Supplementary Section 1. Regulatory Developments on PFAS in Drinking Water 14
Supplementary Section 2. Water Quality and Media Consideration to Calculate Bed Life for IX and GAC 15
Supplementary Section 3. Proposed Framework to Use The QSD Model Based on Local Context 19
Supplementary Table 1. Summary of PFAS occurrence across U.S. drinking water systems 21
Supplementary Table 2. Impact of uncertainty in Freundlich parameters (K and 1/n) on GAC bed life and corresponding system-level cost (USD/m3) and GWP (kg CO2 eq/m3) 24
Supplementary Table 3. Estimated Parameter Values for IX and GAC Bed Life Equations 25
Supplementary Table 4. Water quality of GWs used to develop the bed life for IX using Purofine PFA694E and Dowex PSR-2 26
Supplementary Table 5. Studies used to develop GAC bed life estimates 27
Supplementary References 29

Supplementary Results

Effects of Water Quality on Cost and Environmental Impacts of GAC and IX.

In addition to the baseline analysis for groundwater (GW), economic and environmental outcomes were estimated for surface water (SW) conditions using both granular activated carbon (GAC) and ion exchange (IX) resin for PFAS treatment in drinking water utilities. The influence of TOC on treatment system performance was evaluated across different TOC ranges. PFAS selective IX resins show limited sensitivity to TOC relative to GAC.[1–4] As a result, under the assumptions applied in this study, changing the water source from GW to SW does not affect cost or environmental impact outcomes for IX. In contrast, differences in results are primarily observed for GAC due to the influence of TOC.

The influence of TOC on GAC performance was evaluated for PFOA across TOC ranges of 0.25–0.50, 1.25–1.50, and 2.50–2.75 mg/L for both small (0.03 MGD) and large (75 MGD) utilities (Supplementary Fig. 4). For GAC systems, PFAS removal performance showed strong sensitivity to TOC due to competition between organic matter and PFAS for adsorption sites. For small utilities, the cost at low TOC (0.25–0.50 mg/L) was 1.58 USD/m^3 (median), with a range of [1.45–1.72] USD/m^3 [hereafter, the 5th and 95th percentile range will be shown in brackets following the median value], which is approximately 7% higher than the corresponding baseline GW cost without TOC consideration (Supplementary Fig. 4a). Cost and GWP remained comparable up to 1.50 mg/L. At higher TOC levels (2.50–2.75 mg/L), both cost and GWP increased substantially and exhibited greater uncertainty, reaching 2.18 [1.18–3.56] USD/m^3 and 0.562[0.420–0.825] kg CO_2 eq/m^3 at 0.03 MGD. A similar trend was observed for large utilities. At 75 MGD, cost increased with TOC, from 0.14 [0.13–0.17], 0.16 [0.14–0.19], and 0.66 [0.43–1.89] USD/m^3 at TOC ranges of 0.25–0.50, 1.25–1.50, and 2.50–2.75 mg/L, respectively. At TOC levels above 2.50 mg/L in SW, the cost is approximately four times higher than the baseline GW case. GWP also increased with TOC, reaching 0.290 [0.198–0.542] kg CO_2 eq/m^3 at 2.50–2.75 mg/L, which is approximately 36% higher (median) than the baseline GW value (i.e., 0.212 [0.164–0.270] kg CO_2 eq/m^3). Under elevated TOC conditions, IX showed lower cost and GWP compared to GAC for PFOA.

We further evaluated PFHxA to assess the influence of TOC on short chain carboxylic acids. Under the defined model assumptions, the US EPA GAC bed life equation was applicable up to ≈ 2.0 mg/L TOC for this PFHxA. Therefore, the model was applied across TOC ranges of 0.25–0.50, 1.25–1.50, and 1.75–2.00 mg/L for both small (0.03 MGD) and large (75 MGD) utilities (Supplementary Fig. 3). Results from the kernel density analysis showed that at TOC levels greater than 1.75 mg/L, the cost of GAC (i.e., 1.90 [1.72–2.14] USD/m^3 at 0.03 MGD) exceeds that of IX (i.e., 1.76 [1.61–1.92] USD/m^3 at 0.03 MGD). In contrast, the baseline GW analysis indicated lower costs for GAC compared to IX (Fig. 1). These findings highlight the sensitivity of GAC performance to increasing TOC, particularly as adsorption capacity approaches saturation. Overall, the results demonstrate that contextual parameter (e.g., water quality, especially TOC for GAC and inorganics for IX, directly influences bed life, which in turn affects cost and environmental impacts and can shift the relative performance of treatment technologies.

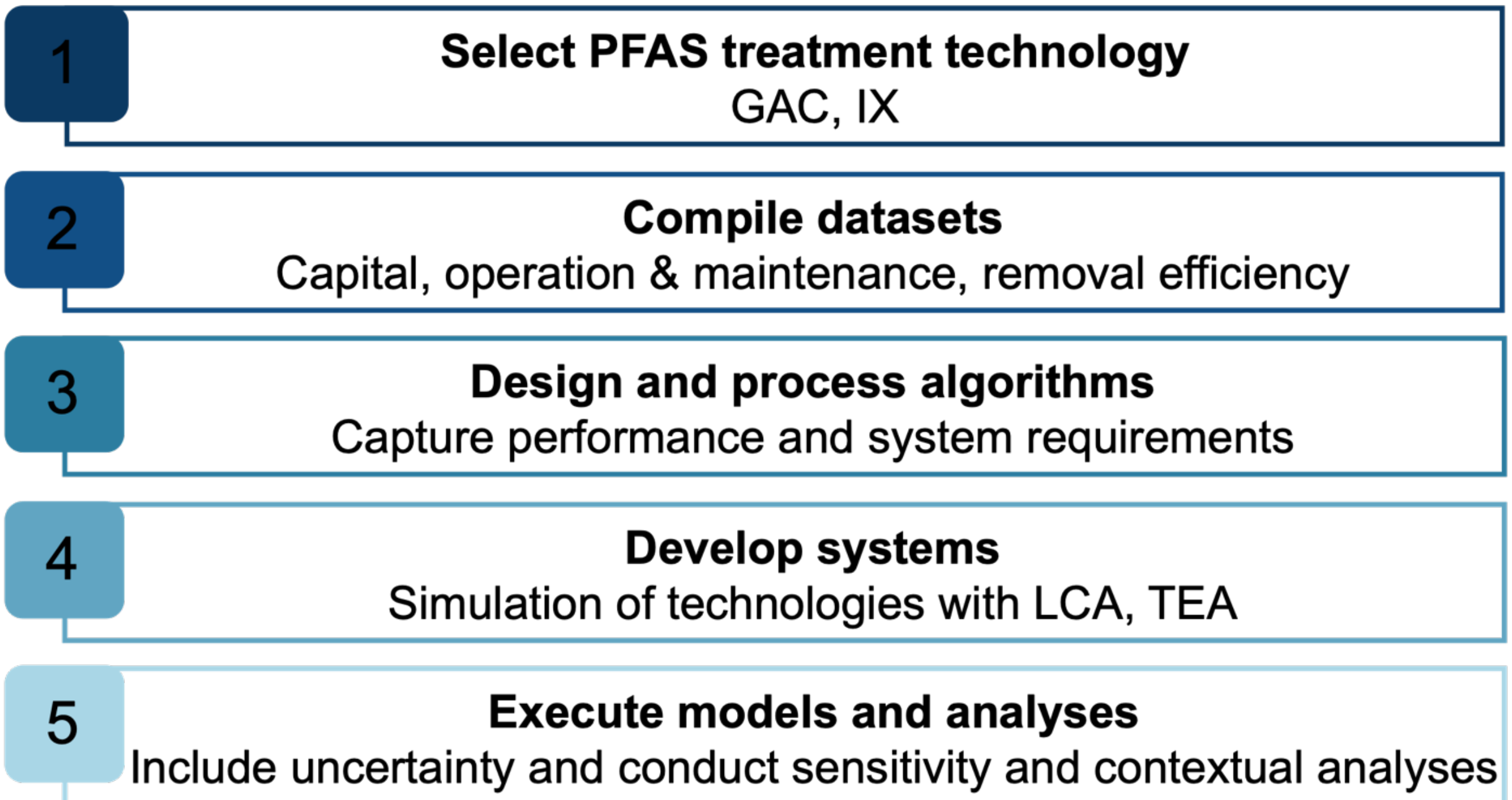

Supplementary Figure 1. Quantitative sustainable design (QSD) methodology applied in this study by selecting PFAS treatment technologies, compiling input datasets, formulating design and process algorithms, constructing system configurations, and executing model simulations and analyses.

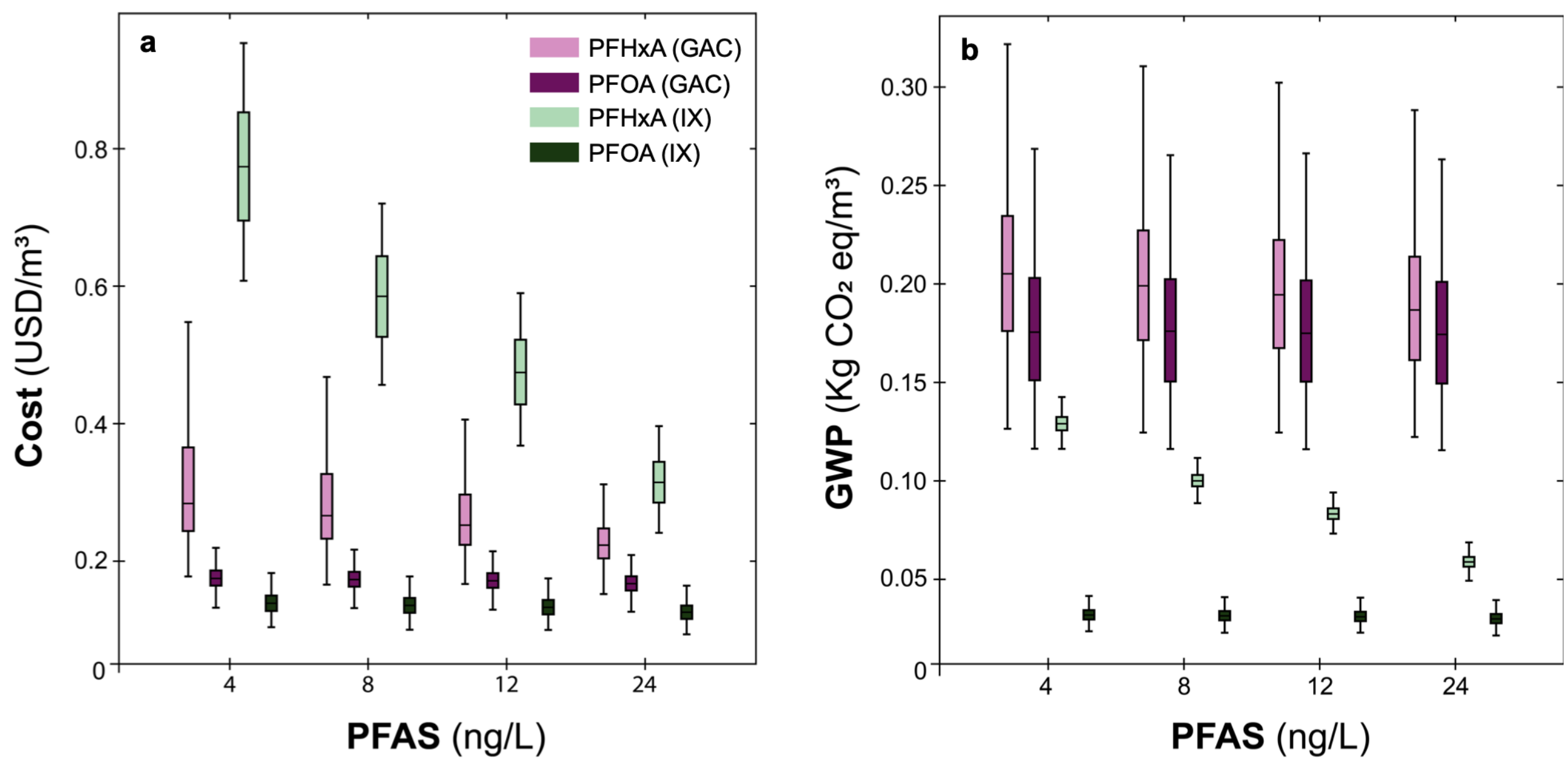

Supplementary Figure 2. Influence of effluent PFAS concentration on cost and GWP for PFAS treatment using IX and GAC at 75 MGD. Treatment cost (a) and GWP (b) under varying effluent PFAS targets (4, 8, 12, and 24 ng/L). Results are shown for PFOA and PFHxA treated using IX (green shades) and GAC (purple shades). The median values are plotted as the center lines, boxes represent the 25th to 75th percentile range, and whiskers show the 5th and 95th percentiles based on 10,000 Monte Carlo simulations. Source data are provided as a Source Data file.

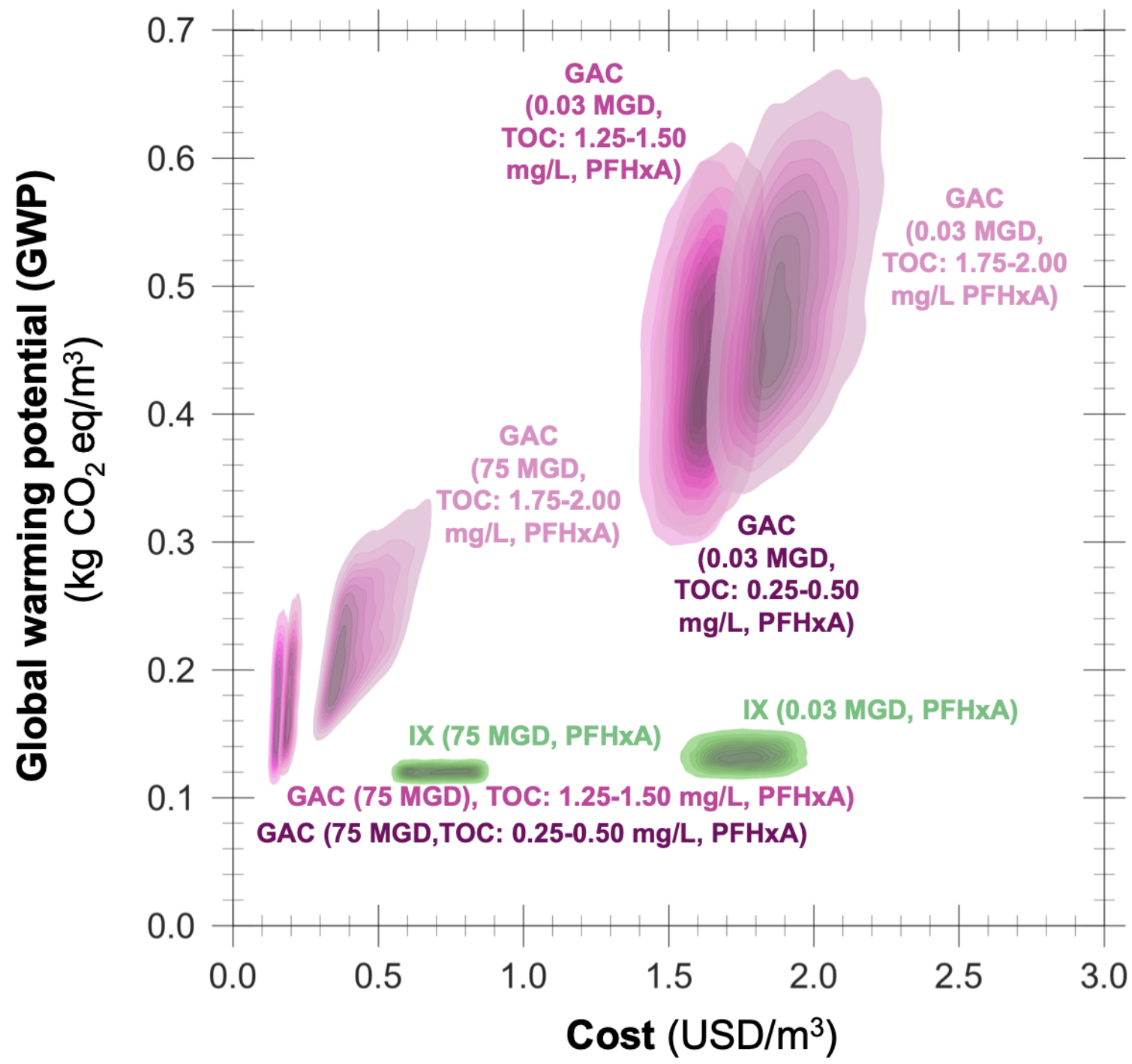

Supplementary Figure 3. The Kernel density maps represent the distribution of estimated GWP emissions and treatment costs for PFAS removal, based on 10,000 Monte Carlo simulations for each scenario. The horizontal axis represents treatment cost (USD/m^3), and the vertical axis represents GWP emissions (kg CO_2 eq/m^3). This plot shows results for IX treatment of PFHxA (dark green) and GAC treatment of PFHxA at TOC of 0.25-0.50 mg/L (dark violet), 1.25-1.50 mg/L (intermediate violet), and 1.75-2.00 mg/L (light violet), implemented at very small (0.03 MGD) and large (75 MGD) utilities. Source data are provided as a Source Data file.

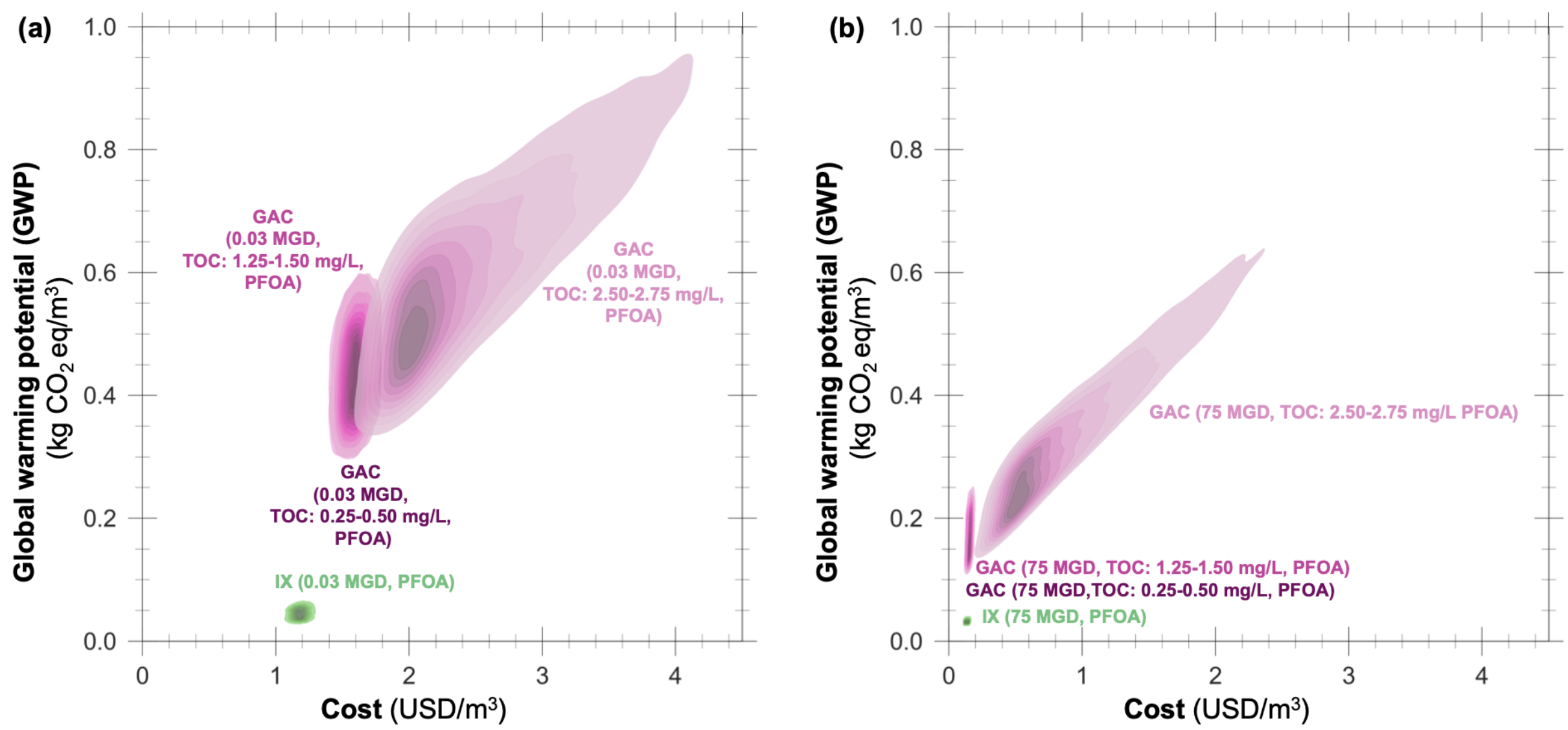

Supplementary Figure 4. The Kernel density maps represent the distribution of estimated GWP emissions and treatment costs for PFAS removal, based on 10,000 Monte Carlo simulations for each scenario. The horizontal axis represents treatment cost (USD/m^3), and the vertical axis represents GWP emissions (kg CO_2 eq/m^3). This plot shows results for IX treatment of PFOA (dark green) and GAC treatment of PFOA at TOC of 0.25-0.50 mg/L (dark violet), 1.25-1.50 mg/L (intermediate violet), and 2.50-2.75 mg/L (light violet), implemented at **a.** very small (0.03 MGD) and **b.** large (75 MGD) utilities. Source data are provided as a Source Data file.

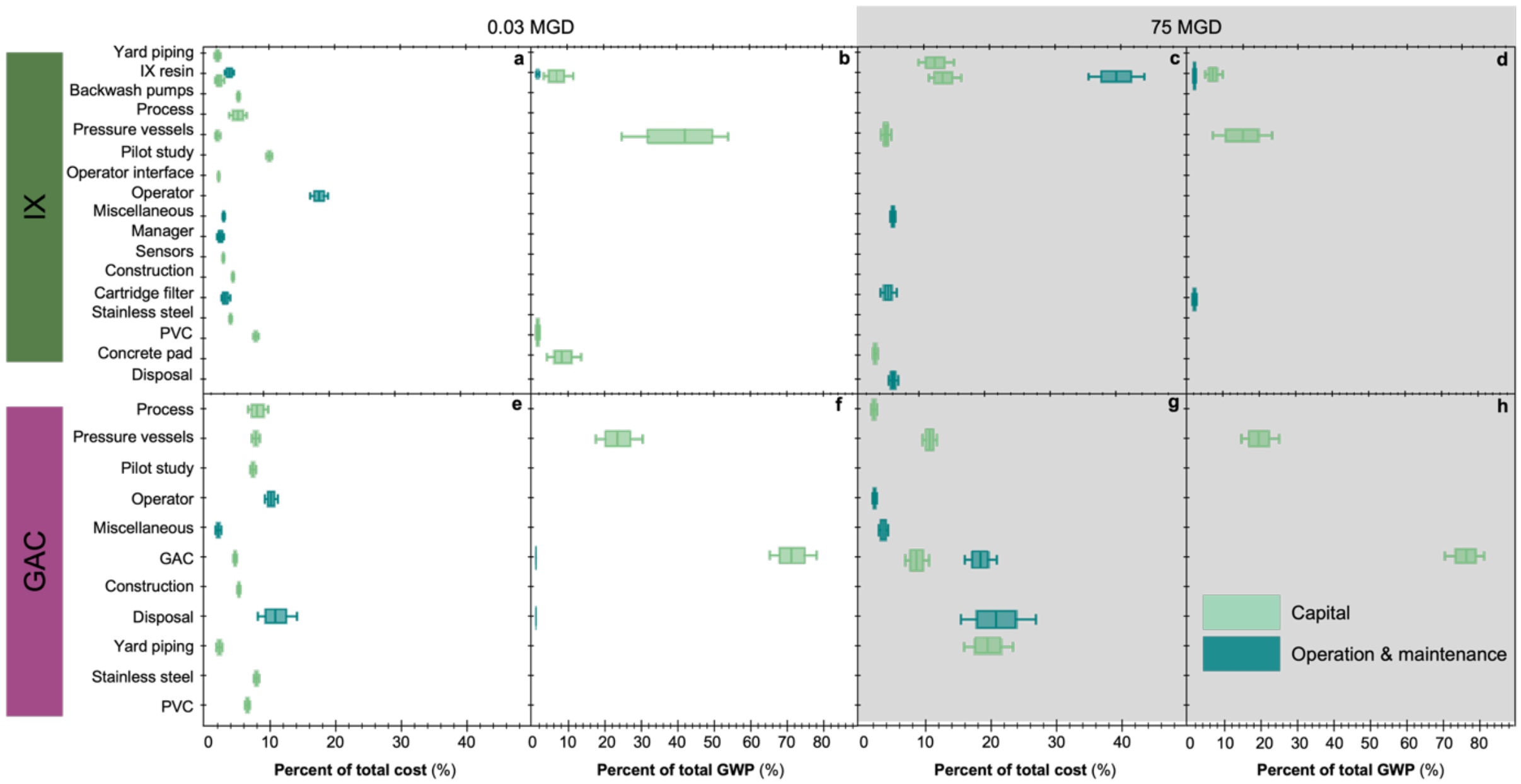

Supplementary Figure 5. Baseline scenarios broken down by unit processes for PFOS treatment using GAC and IX. Percent of total cost is shown for IX treatment in very small utilities (a) and large utilities (c), and for GAC treatment in very small utilities (e) and large utilities (g). Percent of total GWP is presented for IX in very small (b) and large (d) systems, and for GAC in very small (f) and large (h) systems. Cost and GWP are expressed per cubic meter of treated water. First and third columns reflect the relative contributions of capital and OM to median costs, while second and fourth column reflect contributions to median GWP. Electricity contributions were negligible in comparison and are not visible. Box and whisker plots represent the median (center line), interquartile range (25th to 75th percentiles), 10th and 90th percentiles (whiskers), and 5th and 95th percentiles (points), based on 10,000 Monte Carlo simulations. Source data are provided as a Source Data file.

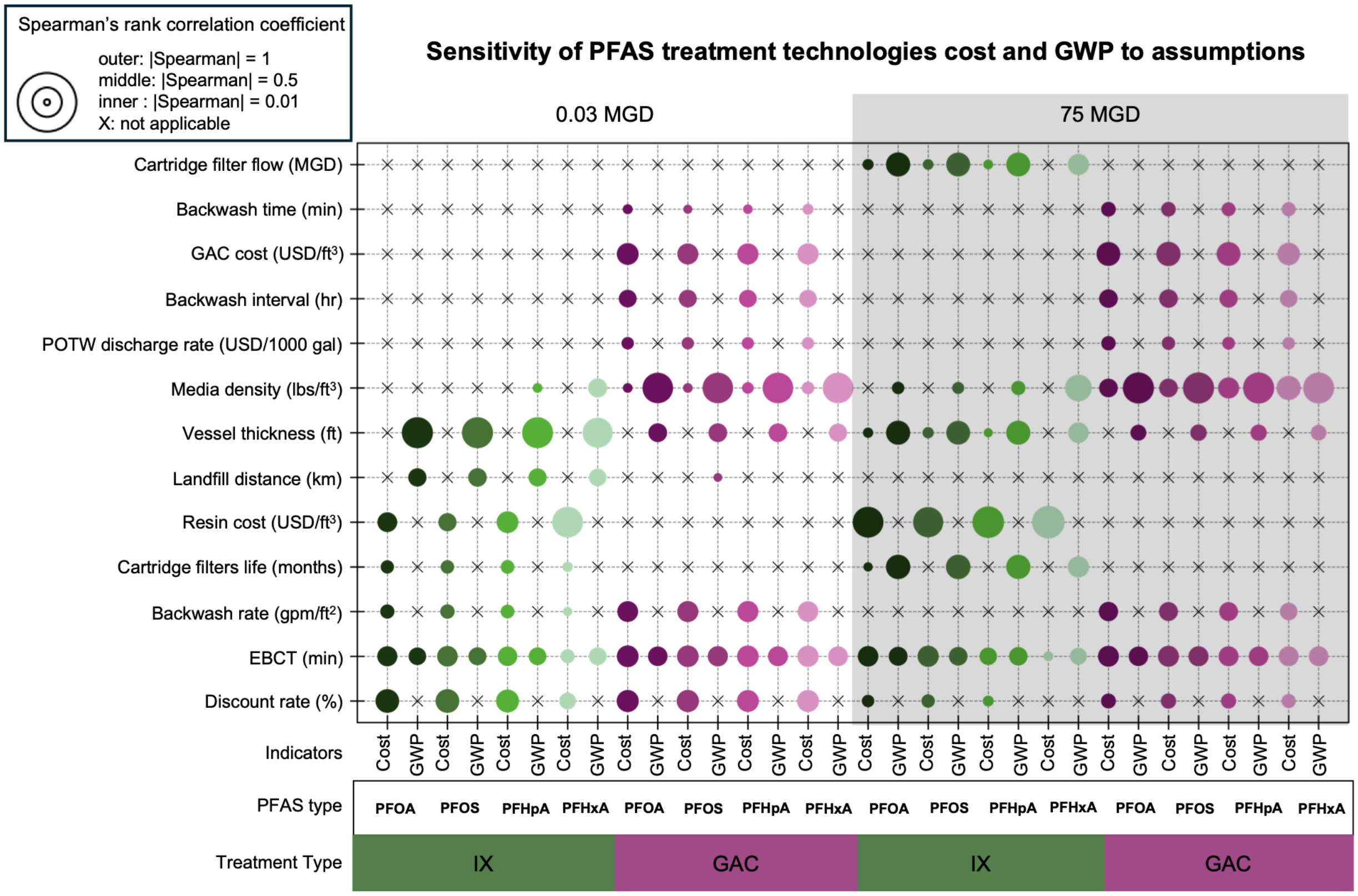

Supplementary Figure 6. Spearman's rank correlation for cost and GWP for GAC and IX systems treating PFOA, PFOS, PFHpA, and PFHxA for very small(0.03 MGD) and large utilities (75 MGD). The key drivers are on the ordinate corresponding with each system's cost and GWP on the abscissa. Bubble size reflects the strength of the correlation, with larger bubbles indicating greater influence; marks indicate parameters not applicable to a given scenario. Source data are provided as a Source Data file.

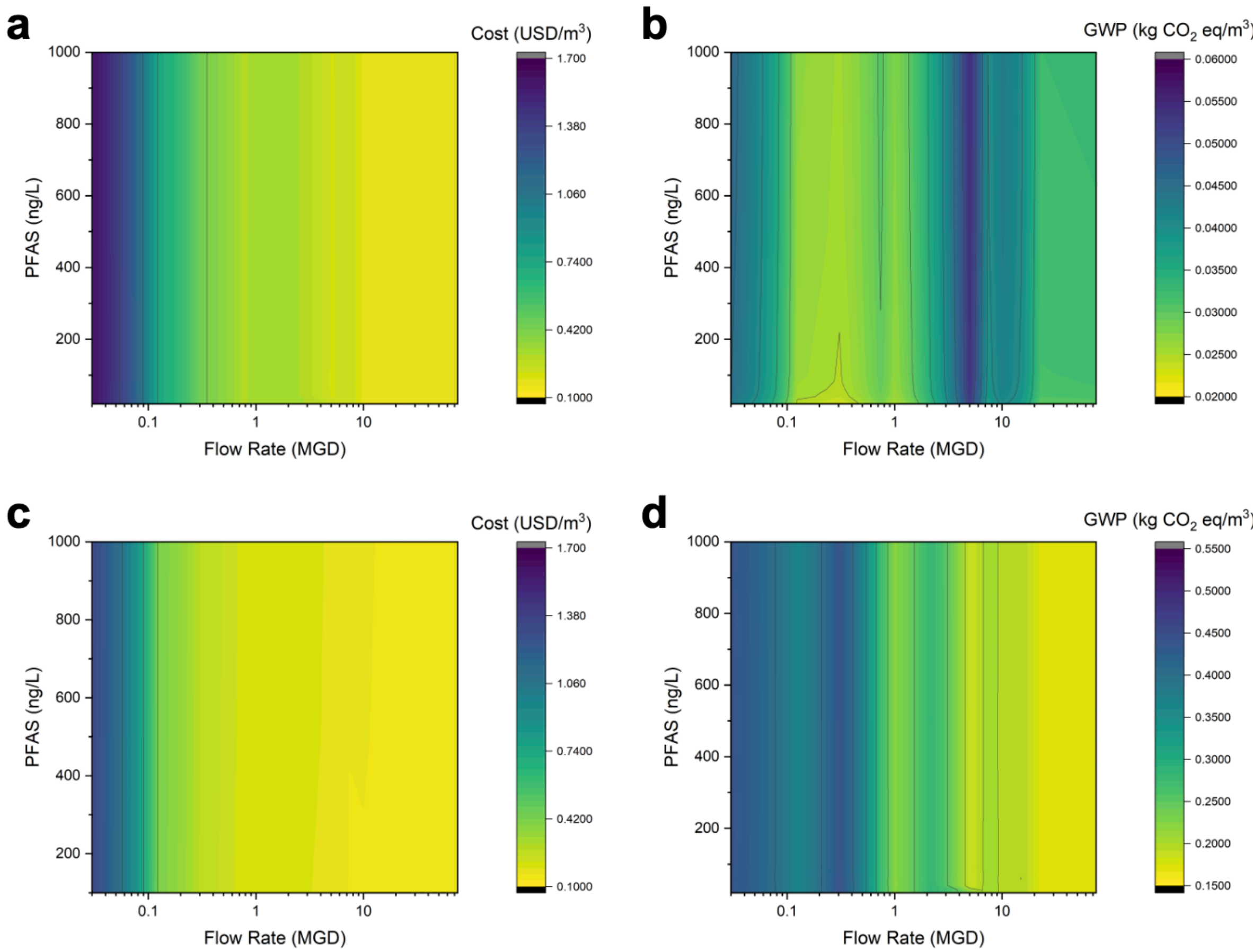

Supplementary Figure 7. Influence of flow rate and PFOA concentration on treatment cost and GWP for IX and GAC technologies. Contour plots showing treatment cost (a) and GWP (b) for IX. Contour plots showing treatment cost (c) and GWP (d) for GAC. Color gradients correspond to treatment cost (USD/m³) and GWP (kg CO_2 eq/m^3), with blue indicating lower values and red indicating higher values. Source data are provided as a Source Data file.

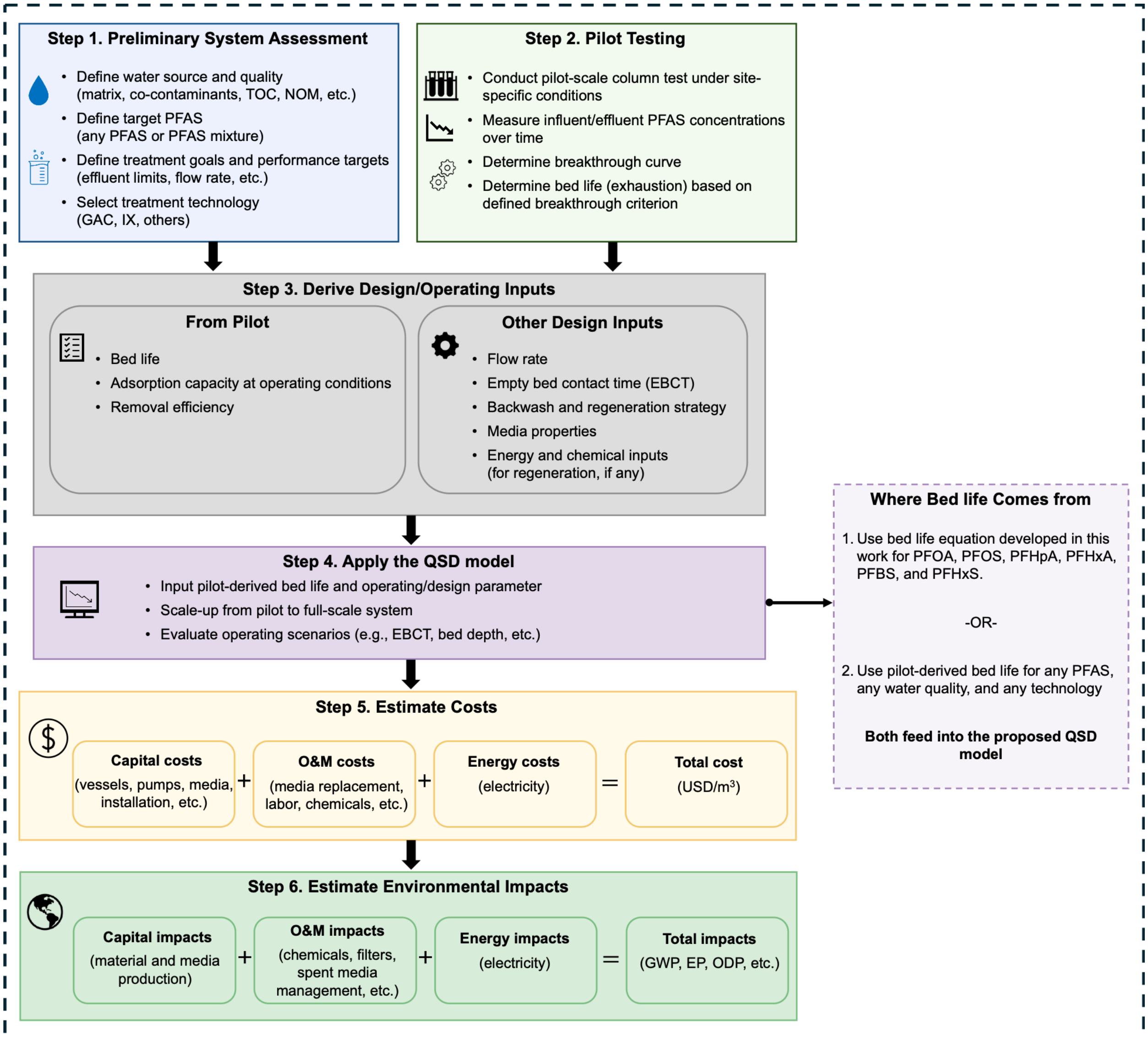

Supplementary Figure 8. Framework for linking pilot-scale performance to cost and environmental impacts of PFAS treatment using QSD model.

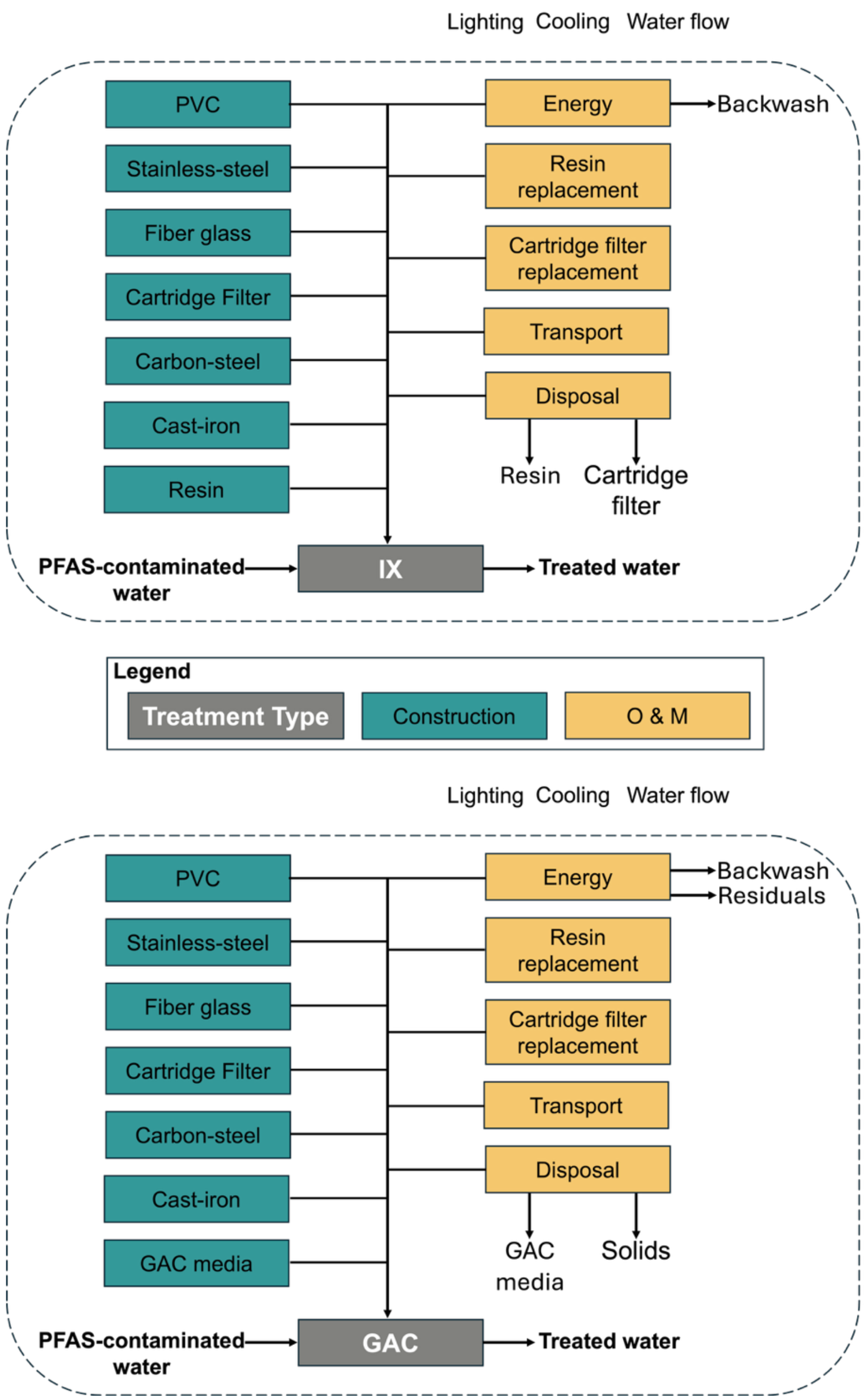

Supplementary Figure 9. System boundaries and foreground inventory for two drinking water treatment systems for PFAS removal: (a) IX and (b) GAC. Each system includes construction components (green) and operation and maintenance inputs (yellow), with the treatment unit itself shown in dark gray. All inventory items represent market activities (i.e., include transportation, where applicable). The dashed box indicates the modeled system boundary; processes beyond this boundary, such as lighting, cooling, and water transfer, are excluded from the analysis.

Supplementary Sections

Supplementary Section 1. Regulatory Developments on PFAS in Drinking Water

In 2024, driven by growing evidence of PFAS contamination, the US EPA finalized its first-ever national drinking water standards for six PFAS compounds-perfluorooctanoic acid (PFOA), perfluorooctanesulfonic acid (PFOS), perfluorohexane sulfonic acid (PFHxS), perfluorononanoic acid (PFNA), hexafluoropropylene oxide dimer acid (HFPO-DA) (GenX), and perfluorobutane sulfonic acid (PFBS)-with mandatory initial monitoring by 2027 and full compliance required by 2029.[5] In 2025, the agency announced plans to extend the compliance deadline for PFOA and PFOS to 2031 to allow drinking water systems additional time to design and implement effective treatment strategies.[6] In addition, the US EPA has announced its intent to rescind the regulations and revisit the underlying regulatory determinations for other PFAS initially proposed. This adjustment is designed to support utilities nationwide, including small and rural systems, in managing these contaminants. Collectively, these actions reflect the agency's acknowledgement that addressing PFAS contamination in drinking water requires realistic timelines together with a sustainable, holistic approach that aligns with the long-term protection of public health and water resources.

Supplementary Section 2. Water Quality and Media Consideration to Calculate Bed Life for IX and GAC

Water quality is a critical factor in selecting appropriate treatment technologies for PFAS removal, as it directly influences both cost and environmental impacts through its effect on bed volume or bed life.[7] In this study, the bed life applied for IX and GAC are derived from empirical datasets and therefore implicitly account for key water quality characteristics. For IX, the bed life model used in this study is based on rapid small-scale column test (RSSCT) data from PFAS-selective resins and incorporated by US EPA into its national cost methodology.[8] These data include two commercially available PFAS-selective resins (i.e., Purofine PFA694E and Dowex PSR-2) tested across six distinct GW matrices with varying pH, alkalinity, dissolved organic carbon (DOC), and competing anions (Table S4). Because the US EPA pooled results across resins and water qualities, the resulting bed life equation reflects central-tendency performance for PFAS-selective IX, rather than product-specific behavior. For GAC, the US EPA bed life equation was derived from 10 water quality conditions across six peer-reviewed studies, explicitly incorporating influent TOC as a controlling parameter due to competitive adsorption between PFAS and organic matter.[8–12] When multiple GACs were evaluated within a study, US EPA selected the best-performing carbon, under the assumption that underperforming media would not be deployed in practice. This approach reflects a design assumption, rather than optimization around specific products. As a result, the GAC model represents upper-bound performance under realistic water quality conditions, rather than a single proprietary carbon.

PFAS selective IX resins show limited sensitivity to total organic carbon (TOC) relative to GAC.[1–4] Performance is instead controlled by the presence of competing inorganic anions, including nitrate, sulfate, bicarbonate, and chloride.[13,14] The IX bed life model follows the US EPA framework and is based on data obtained from RSSCTs rapid small scale column tests designed to simulate full scale operation at an empty bed contact time of 3.3 minutes. The dataset includes six GW sources that capture a range of water quality conditions and PFAS mixtures representative of contaminated drinking water systems. These data were used to develop compound specific bed life relationships using multiple linear regression. The bed life equation (1) for IX applied in this study was recreated from

the US EPA model.[14] In the equation, $BV_{contam,IX}$ represents the bed life for a given PFAS contaminant in bed volumes, $PFAS_{total}$ is the total influent concentration of PFAS compounds (ng/L), and $\%R_{contam}$ is the target % removal of a given PFAS expressed as a decimal (e.g., 0.96).

$$\mathrm{BV}_{contam,IX} = \mathrm{A}_{PFAS} \times \mathrm{PFAS}_{total} + \mathrm{A}_{R,IX} \times \%\mathrm{R}_{contam} + \mathrm{B}_{contam,IX} \quad (1)$$

A_{PFAS}, $A_{R,IX}$, and $B_{contam,IX}$ are regression parameters, and their estimated values are provided in Supplementary Table 3. The model includes a compound specific intercept term ($B_{contam,IX}$) that represents differences among PFAS compounds and implicitly captures the influence of competing inorganic anions. Although nitrate and sulfate were present at concentrations typical of drinking water, their range across the experimental dataset was limited. This limitation prevented explicit inclusion of individual anion concentrations in the final model. As a result, the effects of inorganic constituents are embedded within the empirical relationships for each PFAS, but the model does not allow direct adjustment of specific ion concentrations. The GW quality conditions used to develop the IX bed life model are summarized in Supplementary Table 4. SW systems are typically characterized by higher levels of organic matter.[15] However, as discussed above, for PFAS selective IX resins, performance is governed mainly by competition from inorganic anions rather than organic constituents. As a result, variation in TOC is not expected to substantially influence bed life for IX systems. The current model, therefore, does not include explicit TOC effects for IX bed life. Consequently, under the assumptions applied in this study, the use of GW or SW conditions does not alter cost or environmental impact outcomes for IX. This omission reflects the selectivity of PFAS targeted resins.

For GAC, TOC is explicitly incorporated into the bed life relationship because organic matter competes with PFAS for adsorption sites and reduces carbon capacity.[4,16–18] The GAC bed life model applied in this study follows the US EPA framework and is based on data compiled from six studies representing 10 distinct water quality conditions. Details of the studies used to derive the GAC bed life relationships, including the corresponding TOC conditions, are summarized in Supplementary Table 5. The combined dataset was

used to develop compound-specific bed life relationships through multiple linear regression, consistent with the approach used for IX.

$$\mathrm{BV}_{contam,GAC} = \mathrm{A}_{TOC} \times TOC + \mathrm{A}_{R,GAC} \times \%\mathrm{R}_{contam} + \mathrm{B}_{contam,GAC} \quad (2)$$

In this equation (2), $BV_{contam,GAC}$ represents the bed life for a given PFAS type in bed volumes, TOC denotes the influent concentration of total organic carbon (mg/L), and $\%R_{contam}$ represents the target removal of a given PFAS expressed as a decimal (e.g., 0.96). The regression coefficients A_{TOC}, $A_{R,GAC}$, and $B_{contam,GAC}$ were obtained from the model development, and their estimated values are provided in Supplementary Table 3. The US EPA model is reported to be valid up to a TOC of 3.2 mg/L, corresponding to the upper limit of the underlying dataset.[14] Under the conditions applied in this study, defined by an initial PFAS concentration of 100 ng/L and a target concentration of 4 ng/L, the model remains valid within a lower TOC range. The applicable TOC limit varies by PFAS types (i.e., $B_{contam,GAC}$), with a maximum validity of approximately 2.75 mg/L.

Under constant influent PFAS concentration and target removal, the predicted order of breakthrough for IX is: PFHxA > PFHpA > PFOA > PFBS = PFHxS = PFOS. Here the identical bed lives are predicted for PFBS, PFHxS, and PFOS because their intercept terms (B_{PFBS}, B_{PFHxS}, B_{PFOS}) are not statistically different. For GAC, under constant influent TOC and target removal, the predicted order of breakthrough is: HFPO-DA > PFHxA > PFBS = PFHpA = PFHxS > PFOA > PFOS. Here PFBS, PFHpA, and PFHxS yield identical predicted bed lives due to statistically indistinguishable intercept terms in the US EPA model.

While PFHpA is sometimes described as an intermediate-chain compound, inclusion of these four PFAS captures both functional group and chain-length effects within the constraints of the US EPA treatment model. Although ultrashort-chain PFAS such as PFBA were not explicitly modeled, prior studies report that short-chain carboxylates exhibit similarly rapid breakthrough behavior and reduced adsorption affinity in both GAC and IX systems relative to long-chain PFAS.[19] As a C6 carboxylate, PFHxA captures this lower-affinity, earlier-breakthrough behavior characteristic of ≤C6 perfluoroalkyl carboxylic acids under the US EPA bed life framework. Frequency of detection of PFBA and PFHxA is also comparable in number of samples.[20] Inclusion of

PFHxA therefore provides a representative case for lower-chain carboxylic PFAS within the constraints of available treatment models. While individual compounds may vary slightly in adsorption coefficients, their sustainability implications in the US EPA-based design framework are governed primarily by reduced sorption affinity and shortened bed life relative to long-chain analogues.

Supplementary Section 3. Proposed Framework to Use The QSD Model Based on Local Context

Treatment performance for PFAS removal varies widely across media types, water chemistry, and PFAS mixtures, which limits the direct use of generalized design relationships. To overcome this, a framework is proposed that links pilot-scale performance data to cost and environmental impact estimation using the QSD model.

The framework begins with system characterization, including identification of water quality parameters, PFAS composition, treatment targets, and treatment technologies. Given the strong dependence of treatment performance on site-specific conditions, pilot-scale testing or RSSCTs are used to evaluate media performance under representative conditions. These tests provide key design parameters, most importantly bed life, which reflects the duration of effective contaminant removal before breakthrough.

The derived bed life, along with other operational inputs (e.g., flow rate, EBCT, and system configuration), is then used as an input to the QSD model. The model translates these decision variables, contextual, and technological parameters inputs into two parallel outputs: (i) cost estimates (ii) environmental impacts, evaluated through TEA and LCA. Because both outputs are driven by the same set of performance and design inputs, the framework ensures consistency in evaluating economic and environmental tradeoffs.

This structure allows the model to move beyond reliance on generalized or literature-derived bed life relationships. Instead, users can directly incorporate site- and media-specific performance data obtained from pilot studies. As a result, the framework can be applied to a wide range of treatment scenarios, including different GAC products, IX resins, PFAS mixtures, and water quality conditions. Burkhardt et al. evaluated multiple GAC media (i.e., Calgon F300, Calgon F400, Norit GAC400, Hydrodarco4000, and Evoqua 1230AWC) under two experimental phases (phase I: 125 days, Aug to Dec 2017; phase II: 224 days, Jan to Aug 2018) for treatment to a cumulative PFAS target of 70 ng/L (PFHpA, PFOA, PFNA, PFHxS, PFOS, and GenX). The results show that

Freundlich parameters (K and 1/n) vary substantially across media types and also within the same media under different operating conditions.[21] For instance, Calgon F400 exhibits different bed lives across scenarios representing different treatment positions (i.e., raw influent (C1) and biologically treated water following biologically activated carbon filtration (C3 and C5) and water quality conditions (Supplementary Table 2). These bed life variations, including ±10% uncertainty in K and 1/n, were used as inputs in the proposed framework to estimate cost and environmental impacts. Results reveal that shorter bed life conditions (e.g., 166 days) result in higher cost and GWP, with cost reaching 0.28 [0.24–0.33] USD/m^3 and GWP 0.197 [0.149–0.258] kg CO_2 eq/m^3. In contrast, longer bed life conditions (e.g., 620 days) reduce system burden, with cost decreasing to 0.17 [0.15–0.20] USD/m^3 and GWP to 0.174 [0.129–0.230] kg CO_2 eq/m^3 (Supplementary Table 2). These differences are driven by media replacement frequency and associated material demand, both of which depend on adsorption performance.

Overall, the proposed framework provides a flexible pathway for translating experimentally derived performance into sustainability metrics. This enables more robust, context-specific evaluation of PFAS treatment options and supports informed decision-making based on both cost and environmental considerations.

Supplementary Table 1. Summary of PFAS occurrence across U.S. drinking water systems, listing all 50 states with corresponding cities or towns, population served, PFAS compound(s) detected, measured concentration(s), utility size category, and source water type (e.g., GW and SW)

State	Cities/Towns	Population Served	PFAS Type	Concentration (ppt)	Utility Size*	Water Type
Alabama (AL)	Prattville	45,444	PFOA	19.2	6	SW
	Prattville	45,444	PFOS	22.8	6	SW
	Linden	4,416	PFOA	6.9	5	SW
	Linden	4,416	PFOS	10.8	5	SW
Alaska (AK)	Anchorage	221,351	PFOA	4.6	8	SW
	Barrow	4,900	PFOS	44.1	5	SW
Arizona (AZ)	Gilbert	247,600	PFOS	7.6	8	GW
	Sedona	7,360	PFOA	10.5	5	GW
California (CA)	Pine Valley	300	PFOA	181.4	2	GW
	Pine Valley	300	PFOS	57.2	2	GW
	Fresno	545,716	PFOA	13.5	8	SW
	Fresno	545,716	PFOS	21.5	8	SW
Colorado (CO)	Colorado Springs	27,640	PFOA	10.3	6	SW
	Colorado Springs	27,640	PFOS	11.9	6	SW
	Brighton	7,202	PFOS	5.4	5	GW
Connecticut (CT)	Meriden	58,441	PFOS	7.9	7	SW
	Shelton	72	PFOA	5.6	1	GW
Delaware (DE)	Dagsboro	93	PFOA	6.9	1	GW
	Dagsboro	93	PFOS	8	1	GW
	Dover	73,860	PFOA	4.4	7	GW
Florida (FL)	Deland	291	PFOA	7.6	2	GW
	Miami	2,300,000	PFOS	14	8	GW
Georgia (GA)	Augusta	204,000	PFOS	43.6	8	SW
	Savannah	829	PFOS	9.2	3	GW
Idaho (ID)	Boise	9,000	PFOA	5.8	5	GW
	Boise	9,000	PFOS	9.5	5	GW
	Meridian	142,830	PFOS	4.9	8	GW
Illinois (IL)	Monmouth	3,275	PFOA	9.2	4	GW
	Peoria	137,575	PFOS	4.1	8	SW
Indiana (IN)	Morgantown	15,368	PFOA	6.4	6	GW
	Morgantown	15,368	PFOS	5.3	6	GW
	South Bend	115,000	PFOA	5.2	8	GW
Delaware (DE)	Dagsboro	93	PFOA	6.9	1	GW
	Dagsboro	93	PFOS	8	1	GW
	Dover	73,860	PFOA	4.4	7	GW
Florida (FL)	Deland	291	PFOA	7.6	2	GW
	Miami	2,300,000	PFOS	14	8	GW
Georgia (GA)	Augusta	204,000	PFOS	43.6	8	SW
	Savannah	829	PFOS	9.2	3	GW

State	Cities/Towns	Population Served	PFAS Type	Concentration (ppt)	Utility Size*	Water Type
Idaho (ID)	Boise	9,000	PFOA	5.8	5	GW
	Boise	9,000	PFOS	9.5	5	GW
	Meridian	142,830	PFOS	4.9	8	GW
Illinois (IL)	Monmouth	3,275	PFOA	9.2	4	GW
	Peoria	137,575	PFOS	4.1	8	SW
Indiana (IN)	Morgantown	15,368	PFOA	6.4	6	GW
	Morgantown	15,368	PFOS	5.3	6	GW
	South Bend	115,000	PFOA	5.2	8	GW
Kansas (KS)	Fort Riley	35,784	PFOA	14.1	6	GW
	Great Bend	14,580	PFOS	8	6	GW
Kentucky (KY)	Georgetown	35,714	PFOS	5.9	6	SW
	Cynthiana	7,263	PFOS	14.8	5	SW
Maine (ME)	Sanford	14,328	PFOA	6.2	6	GW
	Sanford	14,328	PFOS	4.4	6	GW
Maryland (MD)	Ellicott City	950	PFOA	9	3	GW
Massachusetts (MA)	Natick	37,006	PFOA	14.3	6	GW
	Natick	37,006	PFOS	37.3	6	GW
	Avon	4,777	PFOS	5.5	5	GW
Michigan (MI)	Kalamazoo	192,992	PFOS	6.6	8	GW
	Dundee	5,400	PFOA	8.2	5	SW
Minnesota (MN)	Waite Park	8,341	PFOS	16.5	5	GW
	Woodbury	82,643	PFOA	12.9	7	GW
Mississippi (MS)	Petal	5,998	PFOA	5.5	5	GW
Missouri (MO)	Camdenton	3,960	PFOS	10.1	5	GW
	St Peters	52,575	PFOA	4.8	7	SW
Montana (MT)	Kalispell	20,008	PFOS	6.6	6	GW
Nebraska (NE)	Plattsmouth	6,503	PFOS	16.9	5	GW
	Grand Island	51,478	PFOS	7.3	7	GW
Nevada (NV)	Elko	23,000	PFOS	29	6	GW
	Reno	442,000	PFOS	13	8	SW
New Hampshire (NH)	Manchester	123,500	PFOA	4.5	8	SW
	Jaffrey	3,800	PFOA	6	5	GW
New Jersey (NJ)	Toms River	123,184	PFOA	4.9	8	GW
	Pennington	555	PFOS	23.8	3	GW
New York (NY)	Mt. Kisco	300	PFOS	94.5	2	GW
	New Hyde Park	120,000	PFOA	5	8	GW
North Carolina (NC)	Lumberton	65,303	PFOA	26	7	GW
	Greensboro	846	PFOA	4.8	3	GW
Ohio (OH)	Cincinnati	750,200	PFOA	5.2	8	SW
Oklahoma (OK)	Coweta	4,251	PFOS	15.3	5	SW
	Broken Arrow	116,330	PFOS	17	8	SW

State	Cities/Towns	Population Served	PFAS Type	Concentration (ppt)	Utility Size*	Water Type
Oregon (OR)	Bend	77,704	PFOA	6	7	SW
	Medford	200	PFOS	9.8	2	GW
Pennsylvania (PA)	Lancaster	120,000	PFOS	4.7	8	SW
	Lehman	875	PFOS	5.2	3	GW
Rhode Island (RI)	Kingston	19,354	PFOA	16	6	GW
	West Greenwich	88,779	PFOA	7.1	7	SW
South Carolina (SC)	Summerville	275	PFOA	5.2	2	SW
	Lexington	47,838	PFOS	5.6	6	SW
South Dakota (SD)	Keystone	4,100	PFOS	10.2	5	GW
Tennessee (TN)	Shelbyville	34,772	PFOA	5.8	6	SW
	Clifton	3,421	PFOS	14.3	5	SW
Texas (TX)	Abilene	125,182	PFOS	25.3	8	SW
	Tye	1,176	PFOS	6.3	4	SW
Utah (UT)	Salt Lake City	360,654	PFOS	7.1	8	SW
	Hildale	6,000	PFOS	13	5	GW
Vermont (VT)	Barre	378	PFOS	4.6	2	GW
Virginia (VA)	Lovingston	6,861	PFOA	10.8	5	SW
	Roanoke	182,700	PFOS	4.8	8	SW
Washington (WA)	Sammamish	79,322	PFOS	9.7	7	SW
	Spokane	4,228	PFOS	6.1	5	GW
West Virginia (WV)	Davisville	34,251	PFOA	29.1	6	GW
	Pennsboro	2,357	PFOA	9.6	4	GW
Wisconsin (WI)	Hayward	57	PFOA	7.3	1	GW
	Hartford	15,805	PFOA	6.5	6	GW
Wyoming (WY)	Riverton	10,330	PFOS	7	6	SW
	Fort Washakie	3,520	PFOA	9.3	5	SW

*Population Served was categorized into Utility Size classes as follows: ≤100 = 1, 101–500 = 2, 501–1000 = 3, 1001–3300 = 4, 3301–10,000 = 5, 10,001–50,000 = 6, 50,001–100,000 = 7, and >100,000 = 8.

Supplementary Table 2. Impact of uncertainty in Freundlich parameters (K and 1/n) on GAC bed life and corresponding system-level cost (USD/m3) and GWP (kg CO2 eq/m3) for treatment to a cumulative 70 ng/L of six PFAS (PFHpA, PFOA, PFNA, PFHxS, PFOS, and GenX), evaluated using the QSD framework.[21]

**Flow rate (19 MGD) (K & 1/n fit)**	**Type of GAC**		
	C1-Calgon F400 (Phase I)(a)	C3-Calgon F400 (Phase I)(b)	C5-Calgon F400 (Phase II)(c)
Bed life (days) (-10% K, +10% 1/n)	187	166	278
Cost (USD/m^3)	0.26 [0.23-0.31]	0.28 [0.24-0.33]	0.22 [0.19-0.26]
GWP (Kg CO_2 eq/m^3)	0.194 [0.145-0.253]	0.197 [0.149-0.258]	0.185 [0.137-0.243]
Bed life (days) (Best Fit K & 1/n)	260	231	423
Cost (USD/m^3)	0.22 [0.20-0.26]	0.24 [0.21-0.28]	0.19 [0.16-0.22]
GWP (Kg CO_2 eq/m^3)	0.186 [0.138-0.244]	0.189 [0.141-0.247]	0.178 [0.132-0.234]
Bed life (days) (+10% K, -10% 1/n)	357	334	620
Cost (USD/m^3)	0.20 [0.17-0.23]	0.20 [0.18-0.24]	0.17 [0.15-0.20]
GWP (Kg CO_2 eq/m^3)	0.181 [0.134-0.236]	0.182 [0.134-0.238]	0.174 [0.129-0.230]

(a) C1 (Phase I) = This column was fed raw inlet water directly from the water treatment plant for 125 days (Aug-Dec 2017)

(b) C3 (Phase I) = This column was fed treated water that had already passed through biologically activated carbon (BAC) filtration prior to entering the GAC column
C5 (Phase II) = This column was also fed treated water that had already passed through BAC filtration, but it was operated during Phase II of the pilot study for 224 days (Jan-Aug 2018)

Supplementary Table 3. Estimated Parameter Values for IX and GAC Bed Life Equations.[14]

Parameter	IX Model Value	GAC Model Value
A_{PFAS}	-6.04	-
A_{TOC}	-	37,932
A_{R}	-198,242	-36,309
B_{PFHxA}	212,867	113,967
B_{PFBS}	439,515	129,357
B_{PFHpA}	319,511	129,357
B_{PFHxS}	439,515	129,357
B_{PFOA}	390,787	139,862
B_{PFOS}	439,515	143,731

Supplementary Table 4. Water quality of GWs used to develop the bed life for IX using Purofine PFA694E and Dowex PSR-2.[8,14]

**Parameters**	**GW-1**	**GW-2**	**GW-3**	**GW-4**	**GW-5**	**GW-6**
pH	7.5	7.6	8.5	8.1	7.9	8.1
Conductivity (μS/cm)	884	964	682	691	496	525
Alkalinity (mg/L as $CaCO_3$)	130	136	200	178	153	133
Turbidity (NTU)	0.26	0.40	0.1	0.07	1.21	0.28
DOC (mg/L)	0.88	0.95	0.21	0.30	0.80	0.40
UV254	0.008	0.01	0.003	0.002	0.010	0.006
Chloride (mg/L)	150	163	40	40	9.3	15.8
Sulfate (mg/L as SO_4)	76	75	183	180	68.3	97.4
Nitrate (mg/L as N)	5.7	4.7	2.0	2.0	1.0	1.2

Supplementary Table 5. Studies used to develop GAC bed life estimates.[14]

Study Type	Number of Water Quality Conditions	Influent TOC (mg/L)	PFAS Compounds Present and Monitored	Types of GAC studied	References
Pilot	3	1.8 to 3.2(a)	PFBA(b), PFPeA(b), PFHxA, PFOA, PFNA(b), PFDA(b), PFBS, PFPeS(b), PFHxS, PFOS, PFNS(b), HFPO-DA(c)	Calgon F400, Calgon F300, Evoqua 1230 AWC, Hydrodarco 4000, Norit GAC 400	21
Pilot	1	2.7(d)	PFPeA(b), PFHxA, PFHpA, PFOA, PFPrS(b), PFBS, PFPeS(b), PFHpS(b), PFHxS, PFOS	Calgon F400, F600, Norit GAC400 and GCN1240	23
CD-RSSCT	1	1.42	PFOA, PFOS	Virgin bituminous coal (12 × 40), Coconut (8 × 30), Coconut (12 × 40), Reactivated bituminous Coal (12 × 40) (e)	24
CD-RSSCT	2	0.53 and 0.78	PFPeA(b), PFHxA, PFHpA, PFOA, PFBS, PFOS	F400, Carbsorb 40, HPC and CMR400	25
CD-RSSCT	1	2.3 to 2.4	PFHpA, PFOA, PFNA(b), PFBS, PFHxS, PFOS	Evoqua 1230CX, Calgon Filtrasorb 600 AR+	26
CD-RSSCT	2	0.4 and 0.88(d)	PFHxA, PFHpA, PFOA, PFBS, PFHxS, PFOS	Filtrasorb 400, Centaur 12 × 40, Norit GAC 400, Hydrodarco 4000, AquaCarb 1230AWC and AquaCarb 1240CAT	8

CD-RSSCT = constant diffusivity rapid small scale column test; TOC = total organic carbon

Notes:

(a) Includes unpublished TOC data from PFAS pilot study

(b) US EPA was not able to develop a breakthrough relationship for this compound

(c) Other non-carboxylate, non-sulfonate PFAS were also present and monitored
(d) Influent DOC
(e) The four GACs tested in the RSSCT study were manufactured and supplied by Calgon Carbon Corporation, Moon Township, Pa.

Supplementary References

1. Smith, S. J. *et al.* Anion Exchange Resin and Inorganic Anion Parameter Determination for Model Validation and Evaluation of Unintended Consequences during PFAS Treatment. *ACS EST Water* **3**, 576–587 (2023).
2. Rahman, M. F., Anderson, W. B., Peldszus, S. & Huck, P. M. Ion-Exchange Treatment of Perfluorinated Carboxylic Acids in Water: Comparison of Polystyrenic and Polyacrylic Resin Structures and Impact of Sulfate on Their Performance. *ACS EST Water* **2**, 1195–1205 (2022).
3. Dixit, F., Barbeau, B., Mostafavi, S. G. & Mohseni, M. PFOA and PFOS removal by ion exchange for water reuse and drinking applications: role of organic matter characteristics. *Environ. Sci. Water Res. Technol.* **5**, 1782–1795 (2019).
4. Chow, S. J. *et al.* Comparative investigation of PFAS adsorption onto activated carbon and anion exchange resins during long-term operation of a pilot treatment plant. *Water Res.* **226**, 119198 (2022).
5. US EPA, O. Per- and Polyfluoroalkyl Substances (PFAS). https://www.epa.gov/sdwa/and-polyfluoroalkyl-substances-pfas (2021).
6. US EPA, O. EPA Announces It Will Keep Maximum Contaminant Levels for PFOA, PFOS. https://www.epa.gov/newsreleases/epa-announces-it-will-keep-maximum-contaminant-levels-pfoa-pfos (2025).
7. Heberling, M. T. *et al.* Linking Water Quality to Drinking Water Treatment Costs Using Time Series Analysis: Examining the Effect of a Treatment Plant Upgrade in Ohio. *Water Resour. Res.* **58**, e2021WR031257 (2022).

8. Zeng, C. *et al.* Removing per- and polyfluoroalkyl substances from groundwaters using activated carbon and ion exchange resin packed columns. *AWWA Water Sci.* **2**, e1172 (2020).
9. Ateia, M., Maroli, A., Tharayil, N. & Karanfil, T. The overlooked short- and ultrashort-chain poly- and perfluorinated substances: A review. *Chemosphere* **220**, 866–882 (2019).
10. Kothawala, D. N., Köhler, S. J., Östlund, A., Wiberg, K. & Ahrens, L. Influence of dissolved organic matter concentration and composition on the removal efficiency of perfluoroalkyl substances (PFASs) during drinking water treatment. *Water Res.* **121**, 320–328 (2017).
11. Appleman, T. D., Dickenson, E. R., Bellona, C. & Higgins, C. P. Nanofiltration and granular activated carbon treatment of perfluoroalkyl acids. *J. Hazard. Mater.* **260**, 740–746 (2013).
12. Berretta, C., Mallmann, T., Trewitz, K. & Kempisty, D. M. Removing PFAS from Water: From Start to Finish. in *Forever Chemicals* (CRC Press, 2021).
13. Cheng, L., Meng, P. & Knappe, D. R. U. Removal of Per- and Polyfluoroalkyl Substances (PFAS) in fixed bed anion exchange reactors: Factors determining PFAS uptake capacity and models predicting PFAS breakthrough. *Water Res.* **268**, 122629 (2025).
14. US EPA. Technologies and Costs for Removing Per- and Polyfluoroalkyl Substances (PFAS) from Drinking Water. (2024).

15. Liu, X. *et al.* Selective Removal of Organic Pollutants in Groundwater and Surface Water by Persulfate-Assisted Advanced Oxidation: The Role of Electron-Donating Capacity. *Environ. Sci. Technol.* **57**, 13710–13720 (2023).
16. Cantoni, B., Turolla, A., Wellmitz, J., Ruhl, A. S. & Antonelli, M. Perfluoroalkyl substances (PFAS) adsorption in drinking water by granular activated carbon: Influence of activated carbon and PFAS characteristics. *Sci. Total Environ.* **795**, 148821 (2021).
17. Qi, Y., Cao, H., Pan, W., Wang, C. & Liang, Y. The role of dissolved organic matter during Per- and Polyfluorinated Substance (PFAS) adsorption, degradation, and plant uptake: A review. *J. Hazard. Mater.* **436**, 129139 (2022).
18. Sadia, M., Beut, L. B., Pranić, M., Wezel, A. P. van & Laak, T. L. ter. Sorption of per- and poly-fluoroalkyl substances and their precursors on activated carbon under realistic drinking water conditions: Insights into sorbent variability and PFAS structural effects. *Heliyon* **10**, (2024).
19. McCleaf, P. *et al.* Removal efficiency of multiple poly- and perfluoroalkyl substances (PFASs) in drinking water using granular activated carbon (GAC) and anion exchange (AE) column tests. *Water Res.* **120**, 77–87 (2017).
20. PFAS Contamination of Drinking Water Far More Prevalent Than Previously Reported | EWG. https://www.ewg.org/research/national-pfas-testing (2020).
21. Burkhardt, J. B. *et al.* Modeling PFAS Removal Using Granular Activated Carbon for Full-Scale System Design. *J. Environ. Eng.* **148**, 04021086 (2022).
22. Rowles, S. lsrowles/PFAS_water_utilities. https://github.com/lsrowles/PFAS_water_utilities (2025).

23. Liu, C. J., Werner, D. & Bellona, C. Removal of per- and polyfluoroalkyl substances (PFASs) from contaminated groundwater using granular activated carbon: a pilot-scale study with breakthrough modeling. *Environ. Sci. Water Res. Technol.* **5**, 1844–1853 (2019).

24. McNamara, J. D., Franco, R., Mimna, R. & Zappa, L. Comparison of Activated Carbons for Removal of Perfluorinated Compounds From Drinking Water. *J. AWWA* **110**, E2–E14 (2018).

25. Park, M. *et al.* Adsorption of perfluoroalkyl substances (PFAS) in groundwater by granular activated carbons: Roles of hydrophobicity of PFAS and carbon characteristics. *Water Res.* **170**, 115364 (2020).

26. Effectiveness of point-of-use/point-of-entry systems to remove per- and polyfluoroalkyl substances from drinking water - Patterson - 2019 - AWWA Water Science - Wiley Online Library. https://awwa.onlinelibrary.wiley.com/doi/10.1002/aws2.1131.
