# Supplementary material for: Nationwide sustainability assessment of PFAS treatment in U.S. water utilities
Source: Nat Commun. 2026 Oct 1;17:11276. doi: 10.1038/s41467-026-77509-w (PMC13720099; doi:10.1038/s41467-026-77509-w)
Supplement: Supplementary file 2 [file 41467_2026_77509_MOESM2_ESM.pdf]

Description of Additional Supplementary Files

Title: Supplementary Data 1
Description: Input assumptions and parameters for the techno-economic analysis (TEA) and life cycle assessment (LCA) of the ion exchange (IX) treatment system, including economic parameters, design parameters, life cycle inventory impact factors, and engineering sizing and cost lookup tables, with parameter distributions and source references.

Title: Supplementary Data 2
Description: Input assumptions and parameters for the techno-economic analysis (TEA) and life cycle assessment (LCA) of the granular activated carbon (GAC) treatment system, including economic parameters, design parameters, life cycle inventory impact factors, and engineering sizing and cost lookup tables, with parameter distributions and source references.
