# Supplementary material for: Nationwide sustainability assessment of PFAS treatment in U.S. water utilities
Source: Nat Commun. 2026 Oct 1;17:11276. doi: 10.1038/s41467-026-77509-w (PMC13720099; doi:10.1038/s41467-026-77509-w)
Supplement: Supplementary file 3 [file 41467_2026_77509_MOESM5_ESM.pdf]

nature portfolio

Peer Review File

Nationwide Sustainability Assessment of PFAS Treatment in U.S. Water Utilities

Corresponding Author: Dr Lewis Rowles III

This file contains all reviewer reports in order by version, followed by all author rebuttals in order by version.

Version 0:

Reviewer comments:

Reviewer #1

(Remarks to the Author)
This is the third time I've reviewed this submission, twice at Nature Water and now at Nature Communications. I can imagine the authors aren't unbelievably thrilled that I've been asked to review their submission again.
I appreciate the significant changes to this work through this review process. While everything isn't as I'd like to see changed they have worked to address most of my concerns. The one remaining significant concern I have is the range of PFAS studied and the effluent goals adopted. To me it's disingenuous to suggest there are over 14,000 PFAS so they shouldn't investigate more. Most readers with any PFAS knowledge will know there are many PFAS however there aren't that many that are regulated. Why choose PFHxA? I don't think it's regulated as a sole PFAS goal anywhere that I know of. It is pretty common in the environment though. There are a few (e.g., maybe less than 10) PFAS that are commonly subject to drinking water guidance. If PFHxA was regulated it would likely have a higher acceptable concentration (i.e., not the same as PFOS and PFOA but higher). This would likely be the same for all shorter chain PFAS (e.g., PFBS is often acceptable at much higher drinking water concentrations). So it is likely unfair to say that PFHxA is a limiting PFAS (e.g., section starting at line 187).
As I've said in all three reviews, PFHxA is marginally short chain. There are other more prominent PFAS that are shorter chain and of concern.
For me to find this submission acceptable for publication the authors should:
- From the outset (ie no buried at the end) acknowledge that they use PFHxA but they likely should look at what would really be considered short chain and problematic. PFHxA would be way easier to remove using a sorption technology compared to PFBA, for example.
- Not somewhere in the SI but in the main text really acknowledge that results like those presented in Figure 2 really depend on the effluent criteria and that assuming the same effluent criteria for PFHxA and PFOA is likely unrealistic. Of course they could look at the range of effluent criteria here.
Many readers won't read the entire manuscript and get these nuances.

(Remarks on code availability)

Version 1:

Reviewer comments:

Reviewer #1

(Remarks to the Author)
Thank you to the authors for their careful consideration of my review comments. I recommend this submission be published with no further modifications.

(Remarks on code availability)

Open Access This Peer Review File is licensed under a Creative Commons Attribution 4.0 International License, which permits use, sharing, adaptation, distribution and reproduction in any medium or format, as long as you give appropriate credit to the original author(s) and the source, provide a link to the Creative Commons license, and indicate if changes were made.
In cases where reviewers are anonymous, credit should be given to 'Anonymous Referee' and the source.
The images or other third party material in this Peer Review File are included in the article's Creative Commons license, unless indicated otherwise in a credit line to the material. If material is not included in the article's Creative Commons license and your intended use is not permitted by statutory regulation or exceeds the permitted use, you will need to obtain permission directly from the copyright holder.
To view a copy of this license, visit https://creativecommons.org/licenses/by/4.0/

Response to Reviewer Comments

Referee: 1

1 Comments to the Author

1.1 This is the third time I've reviewed this submission, twice at Nature Water and now at Nature Communications. I can imagine the authors aren't unbelievably thrilled that I've been asked to review their submission again. I appreciate the significant changes to this work through this review process. While everything isn't as I'd like to see changed they have worked to address most of my concerns. The one remaining significant concern I have is the range of PFAS studied and the effluent goals adopted.
Response to Comment 1.1: We sincerely thank the reviewer for the time, effort, and thoughtful feedback provided throughout three rounds of review. We appreciate the reviewer's continued engagement and suggestions, which have helped strengthen the manuscript. We also thank the reviewer for identifying areas that should be further clarified in the manuscript. We have addressed these comments point-by-point below. We hope that these edits fully address the reviewer's remaining concern and that the manuscript is now suitable for publication.

1.2 To me it's disingenuous to suggest there are over 14,000 PFAS so they shouldn't investigate more. Most readers with any PFAS knowledge will know there are many PFAS however there aren't that many that are regulated.
Response to Comment 1.2: We thank the reviewer for this observation and agree that the rationale for compound selection should be more clearly articulated. To clarify this point, we have removed the discussion in the methodology that justified the study scope based on the large number of PFAS compounds reported in the literature. Instead, we now clarify that the PFAS evaluated in this study were selected based on the compounds parameterized within the US EPA WBS PFAS treatment models. These compounds (i.e., PFOA, PFOS, PFHpA, PFHxA, PFBS, and PFHxS) listed in the WBS PFAS treatment models span both carboxylate and sulfonate functional groups and represent a range of chain lengths commonly considered in PFAS treatment evaluations.

We further clarify that although we studied PFBS and PFHxS in our model, these compounds were not included in the final analysis because they do not provide additional treatment-performance information under the US EPA WBS model parameterization. For GAC, PFBS, PFHpA, and PFHxS share identical bed-life relationships, while for IX, PFBS, PFOS, and PFHxS share identical bed-life relationships. As a result, PFHpA and PFOS were selected as representative compounds for the GAC and IX analyses, respectively, avoiding redundant results while preserving the full range of treatment behaviors.

We also acknowledge that limiting the analysis to PFAS compounds currently parameterized within the EPA WBS models constrains the scope of the study. We have revised the limitations section to explicitly acknowledge that the PFAS evaluated in this study may not fully represent the diversity of PFAS chemistries and treatment behaviors. In particular, additional short-chain PFAS, such as PFBA, should be investigated in future work, as these compounds are generally more challenging to remove using sorption-based technologies and may exhibit different sustainability trade-offs than those reported here.

Edited text in Methodology (Line 586-623, Page 23)
PFAS compounds evaluated in this study were selected based on the availability of compound-specific bed-life relationships within the US EPA WBS treatment models, which provide the engineering basis for technology sizing, cost estimation, and sustainability assessment.[1] This study initially studied six PFAS compounds: PFOA, PFOS, PFHpA, PFHxA, perfluorobutane sulfonic acid (PFBS), and perfluorohexanesulfonic acid (PFHxS). However, PFBS and PFHxS were not included in the final analysis because they do not provide additional treatment-performance information under the WBS models. For GAC, PFBS, PFHpA, and PFHxS share identical bed-life relationships, while for IX, PFBS, PFOS, and PFHxS share identical bed-life relationships.[1] To ensure that the selected PFAS span meaningful variability in treatment behavior, compound selection was guided by the US EPA bed life equations[1] (Supplementary Section 2). Consequently, PFOA, PFOS, PFHpA, and PFHxA were selected to represent the full range of distinct treatment behaviors available within the EPA framework while avoiding redundant simulations. Together, these four PFAS were selected to examine how differences in functional group chemistry and chain length influence treatment performance and sustainability outcomes. PFOA and PFHpA are long-chain carboxylic acids, while PFOS and PFHxA represent long-chain sulfonic acid and shorter-chain carboxylic acid, respectively. These compounds were also selected in alignment with the US EPA's first-ever national drinking water regulation for PFAS, which establishes legally enforceable maximum contaminant levels (MCL) and reflects mounting concern over their persistence, toxicity, and widespread occurrence in US water systems.

Edited text in Limitation (Line 484-495, Page 20)
This study is limited to PFAS compounds currently parameterized within the US EPA WBS treatment models to help the utilities focused in achieving compliance with the current regulatory limits and therefore does not capture the full diversity of PFAS chemistries. While the selected compounds represent both carboxylate and sulfonate functional groups as well as longer and shorter chain PFAS, additional compounds may exhibit different treatment behaviors. In particular, PFAS with short chain lengths (e.g., perfluorobutanoic acid (PFBA)[2]) than those evaluated in this study and ultrashort-chain PFAS (e.g., trifluoroacetic acid (TFA)[3]), are generally more difficult to remove using sorption-based technologies and may exhibit different sustainability trade-offs than those reported here. Future research should expand sustainability assessments to additional PFAS compounds to improve understanding of treatment performance, costs, and environmental impacts across a broader range of PFAS chemistries and regulatory scenarios. Nevertheless, the findings provide valuable insights for utilities currently making PFAS treatment decisions under the evolving US regulatory landscape.

1.3 Why choose PFHxA? I don't think it's regulated as a sole PFAS goal anywhere that I know of. It is pretty common in the environment though. There are a few (e.g., maybe less than 10) PFAS that are commonly subject to drinking water guidance. If PFHxA was regulated it would likely have a higher acceptable concentration (i.e., not the same as PFOS and PFOA

but higher). This would likely be the same for all shorter chain PFAS (e.g., PFBS is often acceptable at much higher drinking water concentrations). So it is likely unfair to say that PFHxA is a limiting PFAS (e.g., section starting at line 187). As I've said in all three reviews, PFHxA is marginally short chain. There are other more prominent PFAS that are shorter chain and of concern. For me to find this submission acceptable for publication the authors should: From the outset (ie no buried at the end) acknowledge that they use PFHxA but they likely should look at what would really be considered short chain and problematic. PFHxA would be way easier to remove using a sorption technology compared to PFBA, for example.

Response to Comment 1.3: We thank the reviewer for this comment. To address this concern, we have revised the manuscript to explicitly acknowledge at the beginning of the results section that PFHxA is at the threshold of the short-chain PFAS (i.e., the short-chain PFAS with the largest carbon chain length) and may not be interpreted as representing the most challenging short-chain treatment scenario. To make the chain-length comparison across both functional groups visible in the main text, the previous Supplementary Fig. 2 (PFOS and PFHpA) has been promoted to main-text Fig. 1b, with the original Fig. 1 now designated Fig. 1a; the remaining supplementary figures have been renumbered accordingly.

In addition, we have revised the manuscript to avoid characterizing PFHxA as a limiting or worst-case PFAS. Instead, PFHxA is presented as an illustrative short-chain compound within the US EPA framework, and the limitations section now explicitly acknowledges the need to evaluate additional shorter-chain PFAS including PFBA and TFA in future sustainability assessments. For further details, please refer to our response to Comment 1.2.

Furthermore, although the present study is limited to PFAS compounds currently parameterized within the US EPA WBS framework, the sustainability assessment framework developed here (Supplementary Fig. 8) can be extended to additional PFAS compounds as treatment-performance data become available. This framework provides a pathway for future studies to evaluate the sustainability implications of a broader range of PFAS chemistries, including shorter-chain PFAS that are not currently represented in the EPA models.

Edited text in Results and Discussion (Line 123-133, Page 6)

We selected PFOA (long-chain, C8)[4,5] and PFHxA (short-chain, C6)[5,6] to compare between the PFAS with same functional groups (e.g., perfluoroalkyl carboxylic acids or PFCAs). In this study, PFHxA serves as a representative short-chain PFAS within the US EPA WBS treatment framework and provides insight into the sustainability implications of treating short-chain PFAS using GAC and IX. However, PFHxA is a relatively longer short-chain compound and may not be interpreted as representing the most challenging short-chain treatment scenario. Among perfluoroalkyl carboxylates, sorption weakens with decreasing chain length, so shorter-chain compounds such as perfluorobutanoic acid (PFBA) are expected to be more difficult to remove than PFHxA and may exhibit different sustainability trade-offs.[2] However, PFBA is not parameterized within the US EPA WBS framework and could not be evaluated here.

1.4 Not somewhere in the SI but in the main text really acknowledge that results like those presented in Figure 2 really depend on the effluent criteria and that assuming the same effluent criteria for PFHxA and PFOA is likely unrealistic. Of course they could look at the

range of effluent criteria here. Many readers won't read the entire manuscript and get these nuances.

Response to Comment 1.4: We thank the reviewer for this important comment and agree many readers could miss these nuances as it was discussed at the end of results previously. To address this concern, we have moved the discussion regarding effluent-target dependence to the beginning of the Results and Discussions section in the main text. We now explicitly acknowledge that the primary analysis (Fig 1-4) assumes a uniform 4 ng/L effluent target across all PFAS to provide a standardized comparison framework for GAC and IX. We further clarify that future regulatory thresholds for short-chain PFAS may differ from those established for PFOA and PFOS. Therefore, PFHxA results should be interpreted as conditional on the assumed treatment target. These revisions ensure that readers are made aware of this important context before interpreting the results presented in Figure1-4.

We have also updated the results that shows how varying effluent PFAS concentrations (i.e., 4, 8, 12, and 24 ng/L) impacts on cost and GWP (Supplementary Fig. 2). These findings provide insight into how future regulatory thresholds may influence treatment sustainability and utility burden. These descriptions are included in here for review.

Added statement in Results and Discussion (Line 138-145, Page 6)

While the primary analysis used a uniform 4 ng/L effluent target to provide a standardized comparison framework for GAC and IX, regulatory thresholds for several PFAS remain under active discussion and may vary across compounds and jurisdictions as time progresses. Future regulatory thresholds for short-chain PFAS (e.g., PFBS, and PFHxA) may differ from those established for PFOA and PFOS. Consequently, the PFHxA results presented here (Fig. 1-4) should be interpreted as conditional on the assumed 4 ng/L treatment target. Less stringent targets would reduce media replacement requirements and lower both costs and GWP (Supplementary Fig. 2).

Edited text in Results and Discussion (Line 421-428, Page 17)

Increasing the allowable effluent concentration from 4 to 24 ng/L reduced treatment costs by 6.25-21.35% for GAC and 24.37-46.23% for IX, while GWP decreased by 2.99-8.97% for GAC and 22.48-54.39% for IX at 75 MGD for PFHxA. For PFHxA, less stringent effluent targets substantially reduced media replacement demand and associated environmental burdens, whereas PFOA showed comparatively smaller changes due to its stronger sorption affinity and longer bed life. These results suggest that future regulatory thresholds may strongly influence treatment sustainability, especially for utilities treating lower-affinity short-chain PFAS.

References

1. US EPA. Technologies and Costs for Removing Per- and Polyfluoroalkyl Substances (PFAS) from Drinking Water. (2024).
2. McCleaf, P. *et al.* Removal efficiency of multiple poly- and perfluoroalkyl substances (PFASs) in drinking water using granular activated carbon (GAC) and anion exchange (AE) column tests. *Water Res.* **120**, 77–87 (2017).
3. Zheng, G., Eick, S. M. & Salamova, A. Elevated Levels of Ultrashort- and Short-Chain Perfluoroalkyl Acids in US Homes and People. *Environ. Sci. Technol.* **57**, 15782–15793 (2023).
4. Xiao, F., Zhang, X., Penn, L., Gulliver, J. S. & Simcik, M. F. Effects of Monovalent Cations on the Competitive Adsorption of Perfluoroalkyl Acids by Kaolinite: Experimental Studies and Modeling. *Environ. Sci. Technol.* **45**, 10028–10035 (2011).
5. Zhang, D. Q., Zhang, W. L. & Liang, Y. N. Adsorption of perfluoroalkyl and polyfluoroalkyl substances (PFASs) from aqueous solution - A review. *Sci. Total Environ.* **694**, 133606 (2019).
6. Zheng, G., Eick, S. M. & Salamova, A. Elevated Levels of Ultrashort- and Short-Chain Perfluoroalkyl Acids in US Homes and People. *Environ. Sci. Technol.* **57**, 15782–15793 (2023).
